# Depth-dependent variability of biological nitrogen fixation and diazotrophic communities in mangrove sediments

**DOI:** 10.1186/s40168-021-01164-0

**Published:** 2021-10-27

**Authors:** Zhiwen Luo, Qiuping Zhong, Xingguo Han, Ruiwen Hu, Xingyu Liu, Wenjun Xu, Yongjie Wu, Weiming Huang, Zhengyuan Zhou, Wei Zhuang, Qingyun Yan, Zhili He, Cheng Wang

**Affiliations:** 1 Environmental Microbiomics Research Center, School of Environmental Science and Engineering, Southern Marine Science and Engineering Guangdong Laboratory (Zhuhai), Sun Yat-sen University, Guangzhou, 510006 China; 2 Institute of Biogeochemistry and Pollutant Dynamics, Swiss Federal Institute of Technology, Zurich (ETH Zurich), Universitätstrasse 16, 8092 Zurich, Switzerland; 3 South China Institute of Environmental Sciences, Ministry of Ecology and Environment, Guangzhou, 510530 PR China

**Keywords:** Diazotroph, Nitrogen fixation rate, Depth-dependent variability, Nitrogen cycling, Microbiome

## Abstract

**Background:**

Nitrogen-fixing prokaryotes (diazotrophs) contribute substantially to nitrogen input in mangrove sediments, and their structure and nitrogen fixation rate (NFR) are significantly controlled by environmental conditions. Despite the well-known studies on diazotrophs in surficial sediments, the diversity, structure, and ecological functions of diazotrophic communities along environmental gradients of mangrove sediment across different depths are largely unknown. Here, we investigated how biological nitrogen fixation varied with the depth of mangrove sediments from the perspectives of both NFR and diazotrophic communities.

**Results:**

Through acetylene reduction assay, *nifH* gene amplicon and metagenomic sequencing, we found that the NFR increased but the diversity of diazotrophic communities decreased with the depth of mangrove sediments. The structure of diazotrophic communities at different depths was largely driven by salinity and exhibited a clear divergence at the partitioning depth of 50 cm. Among diazotrophic genera correlated with NFR, *Agrobacterium* and *Azotobacter* were specifically enriched at 50–100 cm sediments, while *Anaeromyxobacter*, *Rubrivivax*, *Methylocystis*, *Dickeya*, and *Methylomonas* were more abundant at 0–50 cm. Consistent with the higher NFR, metagenomic analysis demonstrated the elevated abundance of nitrogen fixation genes (*nifH/D/K*) in deep sediments, where nitrification genes (*amoA/B/C*) and denitrification genes (*nirK* and *norB*) became less abundant. Three metagenome-assembled genomes (MAGs) of diazotrophs from deep mangrove sediments indicated their facultatively anaerobic and mixotrophic lifestyles as they contained genes for low-oxygen-dependent metabolism, hydrogenotrophic respiration, carbon fixation, and pyruvate fermentation.

**Conclusions:**

This study demonstrates the depth-dependent variability of biological nitrogen fixation in terms of NFR and diazotrophic communities, which to a certain extent relieves the degree of nitrogen limitation in deep mangrove sediments.

Video Abstract

**Supplementary Information:**

The online version contains supplementary material available at 10.1186/s40168-021-01164-0.

## Background

Mangroves are highly productive ecosystems with immense ecological values towards shoreline protection, climate mitigation, and carbon storage [1, 2]. Their high productivity is greatly attributed to the high nitrogen-fixing activity of diazotrophs, which contributes to 40–60% of the total nitrogen required by mangrove ecosystems [3]. However, due to tidal fluctuation and high denitrification rates, mangrove ecosystems are considered nitrogen-limited [4]. Being the main source of nitrogen inputs in mangrove ecosystems, nitrogen fixation has been demonstrated to primarily affect the nutrient status of sediments [5, 6]. Therefore, as the rate-limiting step of nitrogen cycling, nitrogen fixation is particularly important to alleviate the nitrogen limitation of mangrove ecosystems [7, 8].

Diazotrophs are biological engines to fix atmospheric nitrogen into mangrove ecosystems [9]. Early evidence revealed a high rate of biological nitrogen-fixing activity of diazotrophs in surficial mangrove sediments [10], and a wide range of *Proteobacteria* was thought to be the prevalent diazotrophic groups [11]. Since mangrove ecosystems are rich in sulfur [12], specific diazotrophs are expected in such tidal swamp ecosystems. For example, some sulfate-reducing bacteria (i.e., *Desulfobacteraceae*, *Desulfovibrionaceae*, and *Desulfuromonadaceae*) were identified as diazotrophs in mangrove sediments, which indicated their potential roles in the cycling of multiple elements [11, 13]. However, such knowledge was almost inferred from taxonomic information of diazotrophic communities via either 16S rRNA or *nifH* gene amplicon sequencing, and a robust evidence for the activity, diversity, and versatile functions of diazotrophs is still missing in mangrove ecosystems.

Our current understanding of diazotrophic community diversity and structure in the mangrove ecosystems is hitherto mainly limited to the surficial layers (i.e., 0–25 cm) of sediment, where the density of microorganisms is high [2, 14]. Yet, little is known about diazotrophic communities in deeper (>25 cm) mangrove sediments. In fact, surficial and deep sediments have a substantial variation in environmental properties [15], which have been reported to potentially lead to niche differentiation in diazotrophic communities [16]. For example, the lower oxygen concentration in deeper mangrove sediments tended to facilitate the survival of most diazotrophs [17]. In the sediments with higher salinity, certain diazotrophic families (e.g., *Alteromonadaceae* and *Halanaerobiaceae*) adapting to the higher extracellular osmotic pressure accounted for a higher proportion of total diazotrophs [18]. Similarly, in the sediments with lower water content, the diazotrophic members resistant to the decreased osmotic potential increased in abundance [19, 20]. These previous findings indicated that the depth-dependent variability of environmental properties tended to alter the diazotrophic community composition in mangrove sediments. Nevertheless, the relative importance of these physicochemical properties in determining the in-depth profile of diazotrophic community structure in mangrove sediments is still unknown.

A central assumption of the progressive nitrogen limitation is that, without changes in exogenous nitrogen exchange in an ecosystem, increases in plant nitrogen uptake require an enhanced soil nitrogen cycling rate [21]. This indicates a competition between plant nitrogen acquisition and microbiome-mediated nitrogen transformation processes [3, 22]. Nitrogen availability, driven by the balance among various nitrogen transformation processes, was thought to strongly regulate the ecological functions in both terrestrial and aquatic ecosystems [23, 24]. Being a critical connection between land and ocean [25], mangroves are characterized by nitrogen limitation. However, how the biological nitrogen fixation and its downstream processes in nitrogen cycling influence the degree of mangrove nitrogen limitation is still a vacancy. To fill this gap, we investigated the in-depth profile of biological nitrogen fixation and its downstream processes of nitrogen cycling in mangrove sediments, which is crucial for our better understanding of the prevalent nitrogen limitation across wetlands.

In this study, we aimed to investigate how biological nitrogen fixation varied with the depth of mangrove sediments and identify the key factors affecting the in-depth profile of diazotrophic communities. Through the acetylene reduction assay, *nifH* gene amplicon and metagenomic sequencing, we analyzed NFR, diazotrophic communities, and their key functional genes in 100 cm mangrove sediments, with an interval of 10-cm depth. Besides, draft genomes of diazotrophs were constructed to determine their potential metabolic pathways and adaptation strategies. This study reveals the depth-dependent variability of biological nitrogen fixation and diazotrophic communities in mangrove sediments and advances our understanding of nitrogen limitation mechanisms in mangrove ecosystems.

## Methods

### Site description and sampling

The sampling site is located at the Qi’ao Mangrove Wetland Park (22° 26′ 12.28′′ N, 113° 38′ 26.12′′ E) of Guangdong province, China (Additional file 2: Fig. S1), with a mean annual temperature of 22.4°C and annual precipitation of 1700–2200 mm. The irregular semidiurnal tides were on average 0.17 and −0.14 m of high and low tide levels, respectively [26]. We collected these partially air-exposed sediments from the dominant species, *Sonneratia apetala*, in the Qi’ao Mangrove Wetland Park [26]. Three replicate sediment cores were collected in August 2019 using a 1-m long PVC sampling column after ebb. The sediment cores were sliced at 10-cm intervals into 10 depths (0–10, 10–20, 20–30, 30–40, 40–50, 50–60, 60–70, 70–80, 80–90, and 90–100 cm), yielding a total of 30 samples. Sliced sediments were stored in a portable cooler at 4°C and transported back to the laboratory within 24 h. Each sample was then divided into two sub-samples: one was stored at 4°C for physicochemical properties analysis, and the other was kept at −80°C for DNA extraction.

### Physicochemical properties analysis

NFR was measured by acetylene reduction assay [27]. Briefly, fresh sediment (10.0 g) was put into a 100-mL serum vial. The vials were sealed with rubber stoppers, and 10% of the headspace was replaced with pure and fresh acetylene (C_2_H_2_) before they were incubated in dark at 25°C. After incubation for 48 h, 200 μL headspace gas was taken out to measure the concentration of ethylene (C_2_H_4_) by gas chromatograph (HP7890B, Agilent, USA) equipped with a flame ionization detector and a HP-PLOT MoleSieve5A capillary column (30.0 m × 530 μm × 50 μm) (Agilent, USA ), and He was used as a carrier gas [28]. The detailed NFR calculation method applied in this research is shown in Additional file 1.

Ammonia, nitrite, and nitrate were determined by a multimode microplate reader (Varioskan LUX, Thermo Scientific, USA) after extraction from a 2.0-g fresh sediment with 2 M KCl. Fully digestion method was used to extract total irons, and AB-DTAP extraction method was used to extract the available irons from a 0.5-g air-dried sediment separately [29]. All trace elements were determined by an inductively coupled plasma-optical emission spectrometer (ICP-OES, Avio 500, Perkin Elmer, Singapore). A sequential extraction protocol was used for ferrous and ferric ions from a 0.5-g fresh sample [30], and iron content was measured by ICP-OES (Avio 500, Perkin Elmer, Singapore). The water content of sediment was measured by drying a 10.0-g fresh sediment at 105°C to a constant weight. Sediment pH and salinity were measured with a 2.0-g dry sediment in 1:2.5 (sediment/water) and 1:5 (sediment/water) suspension with a pH meter (SevenCompact210, Mettler-Toledo, USA) and a salinity meter (EUTECH SALT6+, Thermo Scientific, USA), respectively.

### DNA extraction

DNA was extracted and purified with 5.0 g sediment by a combined protocol of sodium dodecyl sulfate extraction method (that was modified by grinding and freezing-thawing) [31] and Power Soil DNA Isolation Kit (Mo Bio Laboratories, Carlsbad, California, USA). DNA purity was checked by NanoDrop ND-2000 Spectrophotometer (Thermo Fisher Scientific, MA, USA), and ratios of 260/280 and 260/230 were about 1.8 and above 1.7, respectively. DNA concentrations were quantified by a fluorescent method (Qubit 4 Fluorometer, Thermo Scientific, USA).

### PCR amplification of *nifH* genes and amplicon sequencing

The *nifH* gene was amplified using the specific primer pair *PolF* (5′-TGCGAYCCSAARGCBGACTC-3′) and *PolR* (5′-ATSGCCATCATYTCRCCGGA-3′) with an expected fragment length of approximately 320 bp [32]. Both forward and reverse primers were tagged with an Illumina adapter sequence, a primer pad, and a linker sequence. The reaction system for each sample was 50 μL, including 25-μL Phusion High-Fidelity DNA Polymerase (NEB, Inc., USA), 2-μL forward and reverse phasing primer, 5-μL DNA template, and 16 μL RNase-free Ultrapure water. The amplification was conducted in a BIO-RAD T100™ thermal cycler (Bio-Rad Laboratory, Hercules, USA) under the following conditions: initial denaturation at 94°C for 5 min, followed by 30 cycles of 94°C for 30 s, 55°C for 30 s, and 72°C for 1 min, with a final extension at 72°C for 10 min. PCR products were then purified using AMPure XP Beads Kit (NEB, Inc., USA). Purified DNA was quantified by Quant-iT™ dsDNA HS Reagent (Thermo Fisher Scientific, Inc., USA) and diluted to a concentration of 2 nM before sequencing. Paired-end *nifH* amplicon sequencing was performed using an Illumina Hiseq 2500 sequencer (Illumina, Inc., CA, USA) at Personalbio Biotechnology Co., Ltd. (Shanghai, China).

The quality filtering and pre-processing of raw sequences were performed on Linux and Galaxy pipeline (http://192.168.3.11:8080/). The primers were firstly eliminated by Cutadapt [33]. The low-quality sequences (quality score <20) were removed by Trimmomatic, and then forward and reverse reads were combined using FLASH [34]. Combined sequences of <285 bp and >350 bp were eliminated, and sequences with one or more ambiguous base(s) (“N”) were also removed. The chimeras were identified and eliminated using UCHIME [35]. FrameBot software was used to correct potential frameshifts caused by sequencing errors [36], and only DNA sequences that covered >30% of reference *nifH* protein translations were retained for further analysis. Operational taxonomic units (OTUs) were clustered at a 95% cutoff [37] of similarity level with protein reference sequences by using Quantitative Insights into Microbial Ecology (QIIME) implementation of UPARSE [38]. Taxonomic assignments for *nifH* OTUs were carried out an 80% identity cutoff [39] by searching representative sequences against reference *nifH* sequences with known taxonomic information [40]. For further analysis, all samples were randomly resampled to the smallest individual sample sequencing effort (23,224) as described before [41].

### Shotgun metagenomic sequencing and data analysis

For surficial (0–10 cm), middle (50–60 cm), and deep (90–100 cm) sediment samples, 1 μg of DNA was used for metagenomic sequencing library preparation combined with NEBNext® UltraTM DNA Library Prep Kit for Illumina (NEB, USA) as recommended by the manufacturer. Index codes were added to attribute sequences to each sample. The samples were purified (AMPure XP system), and the libraries were checked using Agilent 2100 Bioanalyzer (Agilent Technologies, CA) and quantified using real-time quantitative PCR. After cluster generation was performed on a cBot Cluster Generation System, paired-end reads (PE150) were performed on the Illumina platform. Low-quality (quality score ≤38, base N >10 bp, the overlap length between adapter and reads >15 bp) paired-end reads were filtered. The metagenomic assembly was performed using MEGAHIT (v1.2.9) at default mode [42]. For assembled metagenomes, MetaGeneMark (v.2.10) was used to predict open reading frames (ORFs). A non-redundant gene catalog (Unigenes) was built using CD-HIT (v.4.5.8) to predict ORFs [43]. Functional annotation was performed using DIAMOND combined with the Kyto Encyclopedia of Genes and Genomes (KEGG) (http://www.genome.jp/kegg/pathway.html) database (release 94.2), and the KOs (KEGG Orthology) were divided into higher KEGG categories and KEGG pathways. Gene abundances were normalized into transcripts per million (TPM) counts. The TPM values could be applied to metagenomes to remove the effects of total read counts and gene lengths when comparing the abundances of genes between samples [44].

### Metagenomic binning and metagenome-assembled genome (MAG) annotation

Genome assembly and binning were performed according to the MetaWRAP pipeline [45]. The sequences were assembled with MEGAHIT (v1.2.9; options: -mink 21 -maxk 141 -step 12) [42] to generate contigs. Genome binning of assembled contigs was done using MetaBAT2 (v2.12.1) [46] and MaxBin2 (v2.2.7) [47], and the resulting bins were consolidated with the Bin_refinement module. The consolidated bin sets were further improved by the Reassemble_bins module to generate MAGs. The quality of MAGs was evaluated with CheckM (v1.0.5). MAGs were analyzed further if their completeness was more than 50% and their contaminations were below 10%. The abundance of each MAG was expressed as genome copies per million reads and calculated with Salmon [48]. Taxonomic assignments of MAGs were performed using the GTDB-Tk (v0.3.2) [49]. Gene prediction for MAGs was performed using prodigal (v2.6.2, default settings), and the predicted genes were further annotated using KAAS (KEGG Automatic Annotation Server) [50]. Additionally, we utilized a custom HMMER as well as the Pfam (release 33.1) and TIGRFAM databases (release 15.0) to search for key metabolic marker genes using hmmsearch and custom bit-score cutoffs [51].

### Statistical analysis

Pairwise correlations among NFR, physicochemical characteristics, depth, and diazotrophic community diversity of all 30 samples were performed by linear regression analysis with GraphPad Prism (v7.0). Pearson’s correlation analysis was performed to assess the relationships between NFR and the relative abundances of diazotrophic genera in SPSS 24.0 (SPSS Inc., USA). The other statistical analyses were conducted using vegan package (v2.5.6), and ggplot2 package (v3.3.2) was utilized to visualize data in R (v4.0.2). The structure of diazotrophic communities was evaluated based on Bray-Curtis distance among 30 samples, and their hierarchical clustering was performed using Bray-Curtis distance and “ward” linkage. We conducted Linear discriminant analysis Effect Size (LEfSe) on the website http://huttenhower.sph.harvard.edu/galaxy to identify discriminative taxonomic differences between two depth groups (0–50 cm vs. 50–100 cm). Mantel test was performed to determine the significant associations between diazotrophic community structure and sediment properties of 30 samples with 9999 permutations using vegan package (v2.5.6). As previously described [52, 53], we performed pairwise comparisons on the functional genes involved in the nitrogen cycling of sediment samples from three depths (M1: 0–10 cm, M2: 50–60 cm, M3: 90–100 cm) using STAMP (v2.1.3; parameters: two-samples analysis, two-sided, Fisher’s exact test, Asymptotic-CC, Benjamini-Hochberg false discovery rate) and screened out the functional genes with significant differences (*p* < 0.05).

We constructed a structural equation model (SEM) to determine the direct and indirect relationships among sediment physicochemical properties, sediment depths, diazotrophic community richness and structure on the in-depth profile of NFR. Water content and salinity of sediments were chosen in SEM, since both of them were significantly related to depth and identified as the significant predictors of the diazotrophic community structure based on the linear regression analysis and Mantel test. SEM can partition direct and indirect effects that one variable might have on another, estimate and compare the strengths of multiple effects, and ultimately provide mechanistic information on the drivers of diazotrophic communities and NFR [54]. SEM analysis was performed via the robust maximum likelihood evaluation method using AMOS 22.0 (AMOS IBM, USA). The SEM fitness was evaluated on the basis of a non-significant chi-square test (*P* > 0.05), the goodness-of-fit index (GFI), the comparative fit index (CFI), and the root mean square error of approximation (RMSEA).

## Results

### In-depth profile of NFR and physicochemical characteristics in mangrove sediments

**Fig. 1:**
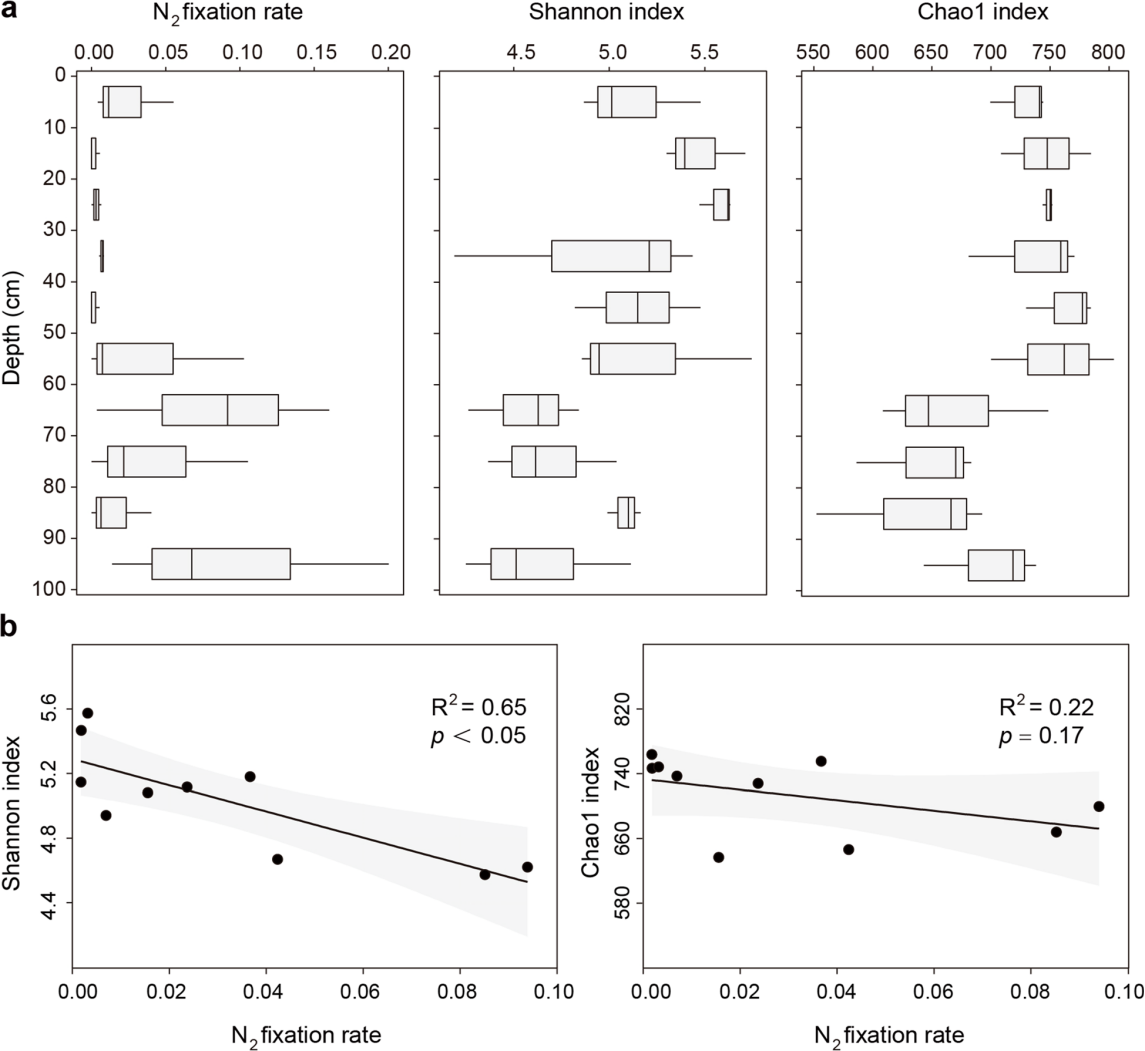
The increased nitrogen fixation rate (NFR) and reduced diversity of diazotrophic communities with depth of mangrove sediments. **a** The in-depth profile of NFR, Shannon index, and Chao1 index of diazotrophic communities. Boxplots depict the 25–75% quantile range of the selected measurements, with the centerline depicting the median (50% quantile). Whiskers show the minimum and maximum values. **b** Relationships between Shannon index and Chao1 index of diazotrophic communities and NFR. NFR was represented as acetylene reduction (nmol C_2_H_4_ g^-1^h^-1^) in this research. Black dots represent the mean values of corresponding indicators for each depth. Black lines and gray shaded areas represent linear regressions and 95% confidence intervals, respectively. *R*^2^ was obtained by linear regression analysis and *p* was obtained by Pearson’s correlation analysis

The NFR fluctuated in the range of 0–0.20 nmol/(g*h), and the average NFR was 0.031 nmol/(g*h) across all 30 samples (Fig. 1a). There was a depth-dependent variability of NFR, which reached a maximum at the depth of 90–100 cm. Compared to the surficial sediments (0–50 cm), the deep sediments (50–100 cm) showed a higher NFR (Fig. 1a). As revealed by the linear regression analysis, we observed a significantly (*R*^2^ = 0.42, *p* < 0.05) positive correlation between NFR and depth of mangrove sediments (Additional file 2: Fig. S2a).

In-depth profile of physicochemical characteristics in mangrove sediments was examined and shown in Additional file 2: Fig. S3. Salinity in mangrove sediments varied from 2.17‰ to 8.90‰ and increased with depth (Additional file 2: Fig. S3f). Conversely, the water content of sediments, with an average of 52%, decreased consistently with depth (Additional file 2: Fig. S3e). The pH, NO_3_^-^ concentration, and total Fe concentration decreased in the surficial sediments (0–50 cm) and subsequently increased with depth (Additional file 2: Fig. S3c, d, g). Among all the measured physicochemical characteristics, only water content and salinity showed significant (*p* < 0.05) linear correlations with depth (Additional file 2: Fig. S2b, c).

### In-depth profile of diazotrophic communities in mangrove sediments

To investigate biotic factors contributing to the increased NFR with depth, we analyzed diazotrophic communities in mangrove sediments by sequencing *nifH* gene amplicons. A total of 2,253,352 high-quality *nifH* sequences were obtained, which were clustered into 974 OTUs and 58 genera after trimming (Additional file 2: Table S1). Notably, we observed a depth-dependent variability of diazotrophic communities in mangrove sediments. The diazotrophic community diversity (Shannon index) and richness (Chao1 index) in depths below 60 cm were lower than those in upper layers (Fig. 1a), and both indices showed significantly (*p* < 0.05) negative correlations with the depth of mangrove sediments (Additional file 2: Fig. S2d, e). Furthermore, the Shannon index of diazotrophic communities, not Chao1 index, showed a significantly (*p* < 0.05) negative relationship with NFR (Fig. 1b). This indicated that the depth-dependent variability of NFR in mangrove sediments was closely related to the shifts in diazotrophic diversity metrics that combined species richness and evenness (i.e., Shannon index).

### Diazotrophs associated with increased NFR at depth

**Fig. 2:**
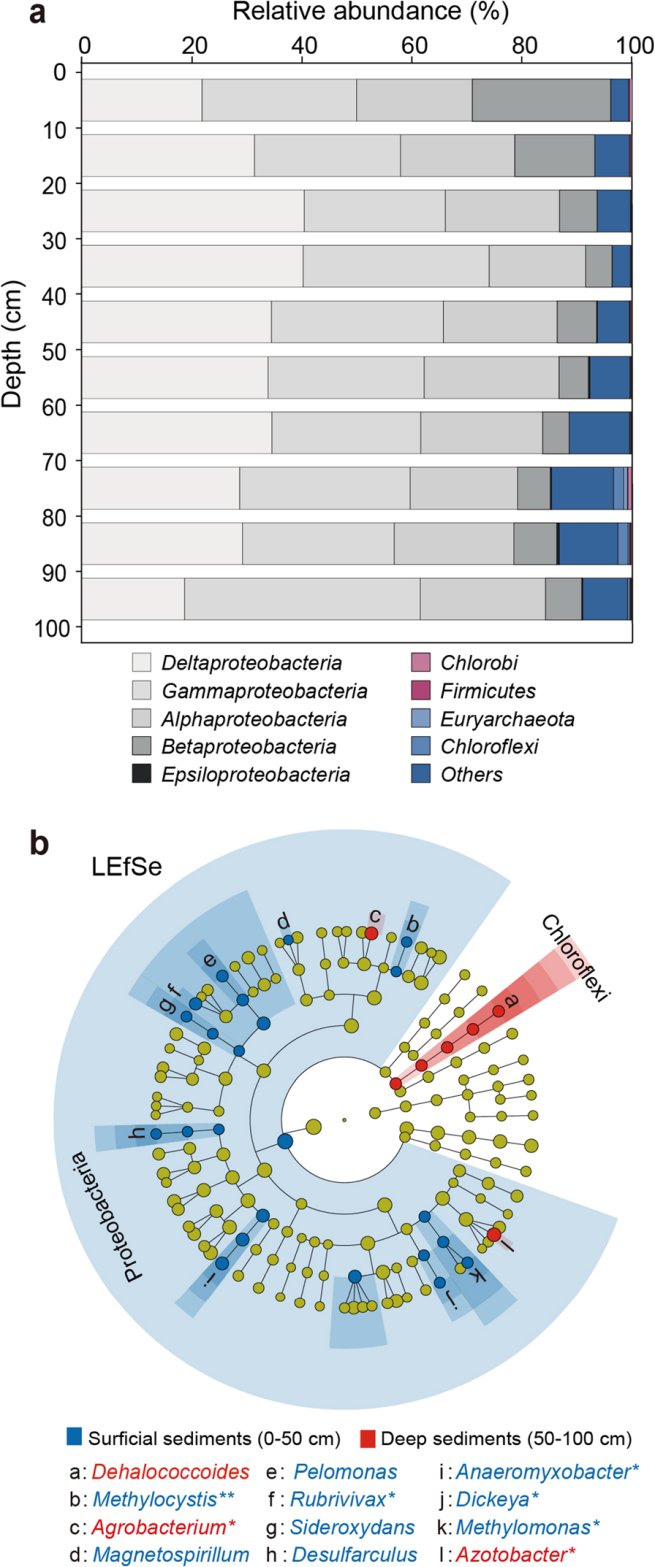
Diazotrophic community composition and specific diazotrophic taxa associated with NFR in mangrove sediments. **a** Taxonomic composition of diazotrophic communities across 10 depths of mangrove sediments. Bar width indicates relative abundance of OTUs from different taxa. **b** Taxonomic cladogram based on linear discriminant analysis (LDA-score >2.0) combined with effect size measurements (LEfSe), which classify discriminative taxa between surficial sediments (0–50 cm, blue) and deep sediments (50–100 cm, red). Moving from inside to outside, cladograms depict domain, phylum, class, order, family, and genus levels. Taxa with non-significant differences are represented as yellow circles. In legend, red * represents the diazotrophic genus having significantly positive correlation with NFR, while blue * represents the diazotrophic genus showing significantly negative correlation with NFR. *p < 0.05; **p < 0.01

Taxonomic analysis showed that bacteria (92.53%) dominated the diazotrophic communities in mangrove sediments, and a few archaea (such as *Methanomicrobia* within the *Euryarchaeota*) (0.18%) were also detected as diazotrophs (Fig. 2a). At the phylum level, *Proteobacteria* was the most prevalent diazotrophs in mangrove sediments, accounting for 91.73% of the total diazotrophs. Among *Proteobacteria*, *Deltaproteobacteria* occupied the largest proportion with an average relative abundance of 31.39%, followed by *Gammaproteobacteria* (30.20%) and *Alphaproteobacteria* (21.11%) (Fig. 2a).

As hierarchical clustering identified 50 cm as partitioning depth where the diazotrophs were generally clustered into two groups (Additional file 2: Fig. S4), we applied the LEfSe to examine the diazotrophic taxa whose abundance was significantly higher in sediment depths above or below 50 cm (Fig. 2b). Results showed that most of the diazotrophs enriched in surficial sediments (above 50 cm) belonged to *Proteobacteria*, including deltaproteobacterial *Anaeromyxobacter* and *Desulfarculus*, gammaproteobacterial *Methylomonas* and *Dickeya*, alphaproteobacterial *Methylocystis* and *Magnetospirillum*, and betaproteobacterial *Rubrivivax*, *Pelomonas,* and *Sideroxydans*. Conversely, alphaproteobacterial *Agrobacterium*, gammaproteobacterial *Azotobacter*, and *Dehalococcoides* within *Chloroflexi* appeared to enrich in deep sediments (below 50 cm) (Fig. 2b).

To further examine whether these deep sediment-specific diazotrophs played major roles in biological nitrogen fixation, we performed Pearson’s correlation analysis between NFR and abundances of diazotrophic genera in mangrove sediment profiles (Additional file 2: Table S2). Specially, two deep sediment-specific diazotrophic genera were positively correlated with NFR, namely *Agrobacterium* (*r* = 0.73, *p* < 0.05) and *Azotobacter* (*r* = 0.48, *p* < 0.05) (Fig. 2b, Additional file 2: Table S2). *Azotobacter* had a higher average relative abundance (11.92%) than *Agrobacterium* (3.28%) (Additional file 2: Table S3). Considering these taxa significantly correlated with NFR and their nitrogen-fixing capacity previously reported [55, 56], we assumed that both *Agrobacterium* and *Azotobacter* contributed to the increased NFR with depth of mangrove sediments. Meanwhile, among 9 diazotrophic genera enriched in surficial sediments, *Anaeromyxobacter* (*r* = −0.73, *p* < 0.05), *Rubrivivax* (*r* = −0.71, *p* < 0.05), *Methylomonas* (*r* = −0.64, *p* < 0.05), *Dickeya* (*r* = −0.65, *p* < 0.05), and *Methylocystis* (*r* = −0.77, *p* < 0.01) showed negative correlations with NFR (Fig. 2b, Additional file 2: Table S2).

### Relationships among sediment physicochemical characteristics, diazotrophic communities, and NFR

Mantel test was performed to quantify the correlations between diazotrophic communities and environmental factors. Result showed that salinity, pH, Fe^3+^ concentration, water content, and NH_4_^+^ concentration exhibited significant (*p* < 0.05) correlations with diazotrophic communities. Among them, salinity showed a much more significant (Mantel *r* = 0.48, *p* < 0.01) correlation (Additional file 2: Table S4), potentially revealing its important role in driving the depth-dependent variability of diazotrophic communities in mangrove sediments.

**Fig. 3:**
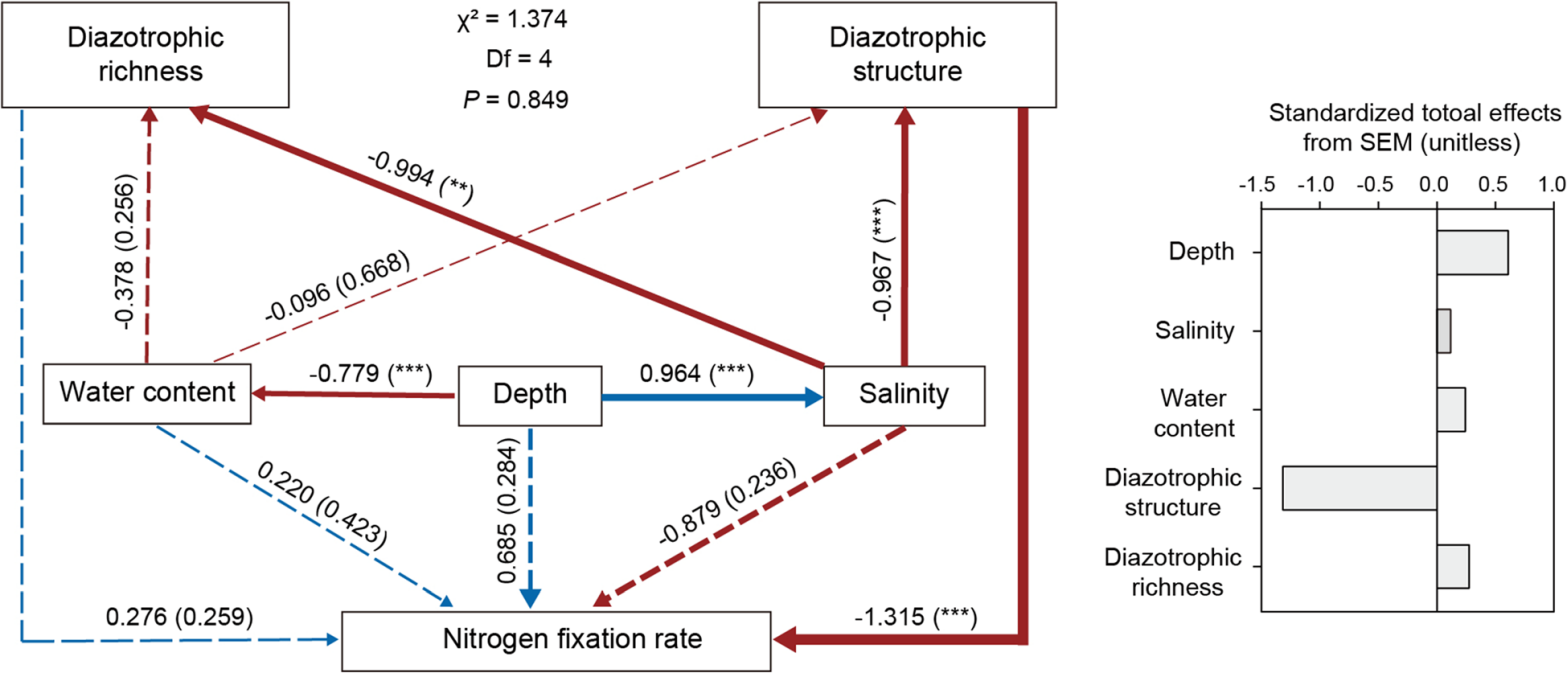
Structural equation modeling (SEM) illustrating the direct and indirect effects of depth, sediment properties (water content and salinity), and molecular attributes (diazotrophic community richness and structure) on NFR. Continuous and dashed arrows represent the significant and non-significant relationships, respectively. Arrows indicate the hypothesized direction of causation. Blue and red arrows indicate positive and negative relationships, respectively. The numbers adjacent to arrows are standardized path coefficients proportional to thickness of the lines, with *p* values in the brackets. Significance levels are denoted with **p < 0.01; ***p < 0.001. Standardized total effects (direct plus indirect effects) calculated by the SEM are displayed beside the SEM. The hypothetical model fits our data well as suggested by the goodness-of-fit statistics: chi-square = 1.374, degrees of freedom = 4, probability level = 0.849, GFI = 0.951, CFI = 1.000, and RMSEA = 0.000

Further, we used SEM to quantify the contribution of each potential influential factor (including depth, water content, salinity, diazotrophic community richness and structure) to the increased NFR (Fig. 3). Consistent with the linear regression analysis (Additional file 2: Fig. S2b, c) and Mantel test (Additional file 2: Table S4), the depth showed a directly positive effect on salinity and a directly negative effect on water content, and salinity exerted a significant effect on the diazotrophic community structure (Fig. 3). Among all the observed variables in the model, diazotrophic community structure was the prominent factor that directly influenced NFR, although depth could indirectly influence NFR by strongly affecting sediment salinity (Fig. 3). Collectively, these results indicated that salinity-driven diazotrophic community structure played an important role in determining the in-depth profile of NFR in mangrove sediments.

### In-depth profile of biological nitrogen fixation and its downstream processes of nitrogen cycling in mangrove sediments

**Fig. 4:**
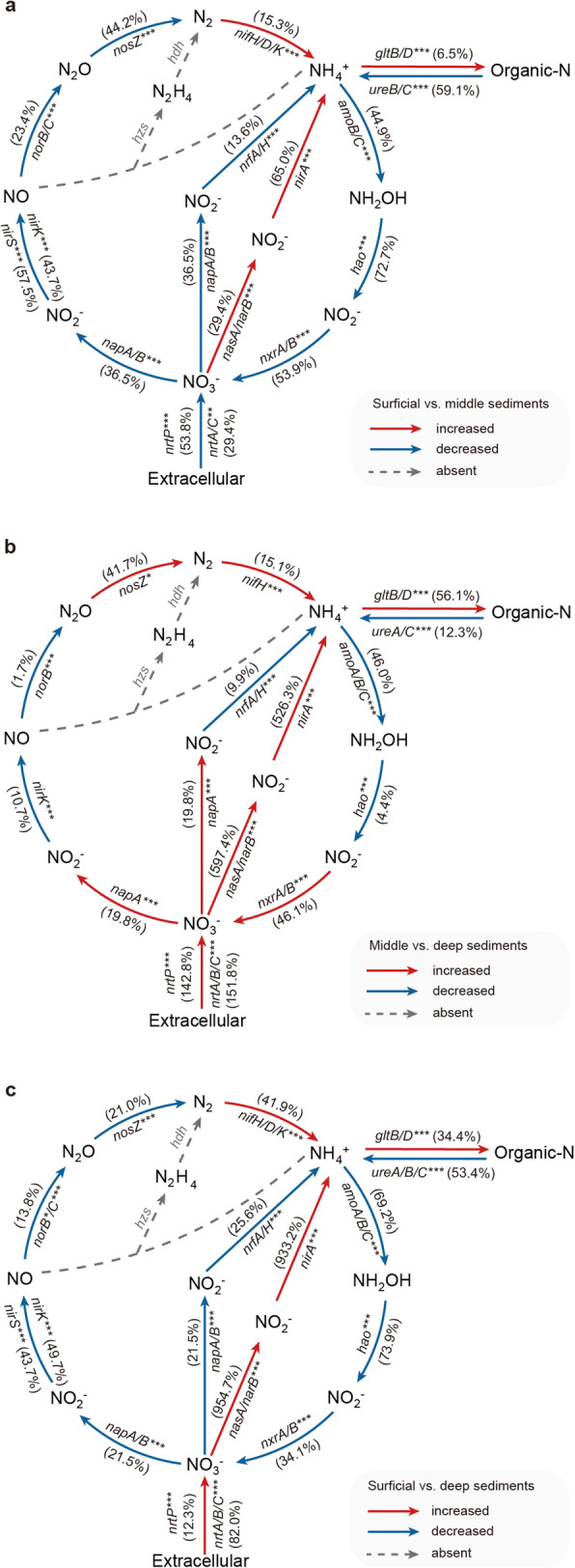
In-depth profile of genes related to nitrogen cycling processes in mangrove sediments. **a–c** The shifts in functional gene abundance in surficial vs. middle sediments (**a**), middle vs. deep sediments (**b**), and surficial vs. deep sediments (**c**). Arrows indicate the directions of reaction. Red arrows indicate that the gene abundance increased from the surficial sediment to middle/deep sediments, and blue arrows indicate a decreasing trend. The genes adjacent to arrows are representative functional genes for each process, and the numbers in the brackets mean the increased or decreased percentages of gene abundance. *p < 0.05; **p < 0.01; ***p < 0.001

We proposed an in-depth schema to illustrate metabolic potentials for various nitrogen cycling processes based on key N-cycling functional genes across the surficial (0–10 cm), middle (50–60 cm), and deep (90–100 cm) sediments (Fig. 4). Notably, a total of eight pathways consistently revealed a depth-dependent variability in terms of functional gene abundances (Fisher’s exact test, *p* < 0.05), including nitrogen fixation, nitrification, denitrification, dissimilatory nitrate reduction to ammonium (DNRA), assimilatory nitrate reduction, ammonia assimilation, nitrate assimilation, and organic *N* decomposition.

Consistent with the trend of diazotrophic activities (NFR), the abundance of gene clusters for nitrogen-fixing (*nifH/D/K*) increased with depth. Compared to that in surficial sediments (M1: 0-10 cm), the abundance of nitrogen fixation genes in deep sediments (M3: 90–100 cm) increased by 41.9% (Fig. 4c). Such an increasing trend also occurred in ammonia assimilation and assimilatory nitrate reduction (Fig. 4). Particularly, from the surficial layers to deep sediments, the functional genes (*nasA*, *narB*, and *nirA*) involved in assimilatory nitrate reduction remarkably increased by 1.5, 17.6, and 9.3 times, respectively (Fig. 4c). By contrast, the abundance of functional genes involved in nitrification (*aomA*, *amoB*, *amoC*, and *hao*), denitrification (*nirK*, *nirS*, *norB*, and *norC*), DNRA (*nrfA* and *nrfH*), and organic *N* decomposition (*ureA*, *ureB,* and *ureC*) significantly (*p* < 0.05) decreased with depth (Fig. 4). Taking the rate-limiting process of denitrification as an example, the abundance of *napA/B* decreased by 21.5% from the surficial layers to deep sediments (Fig. 4c). Overall, these functional gene patterns showed that both biological nitrogen fixation and its downstream processes of nitrogen cycling in mangrove sediments exhibited a depth-dependent variability with divergent trends.

### Versatile functions and adaptation strategies of diazotrophic MAGs

**Fig. 5:**
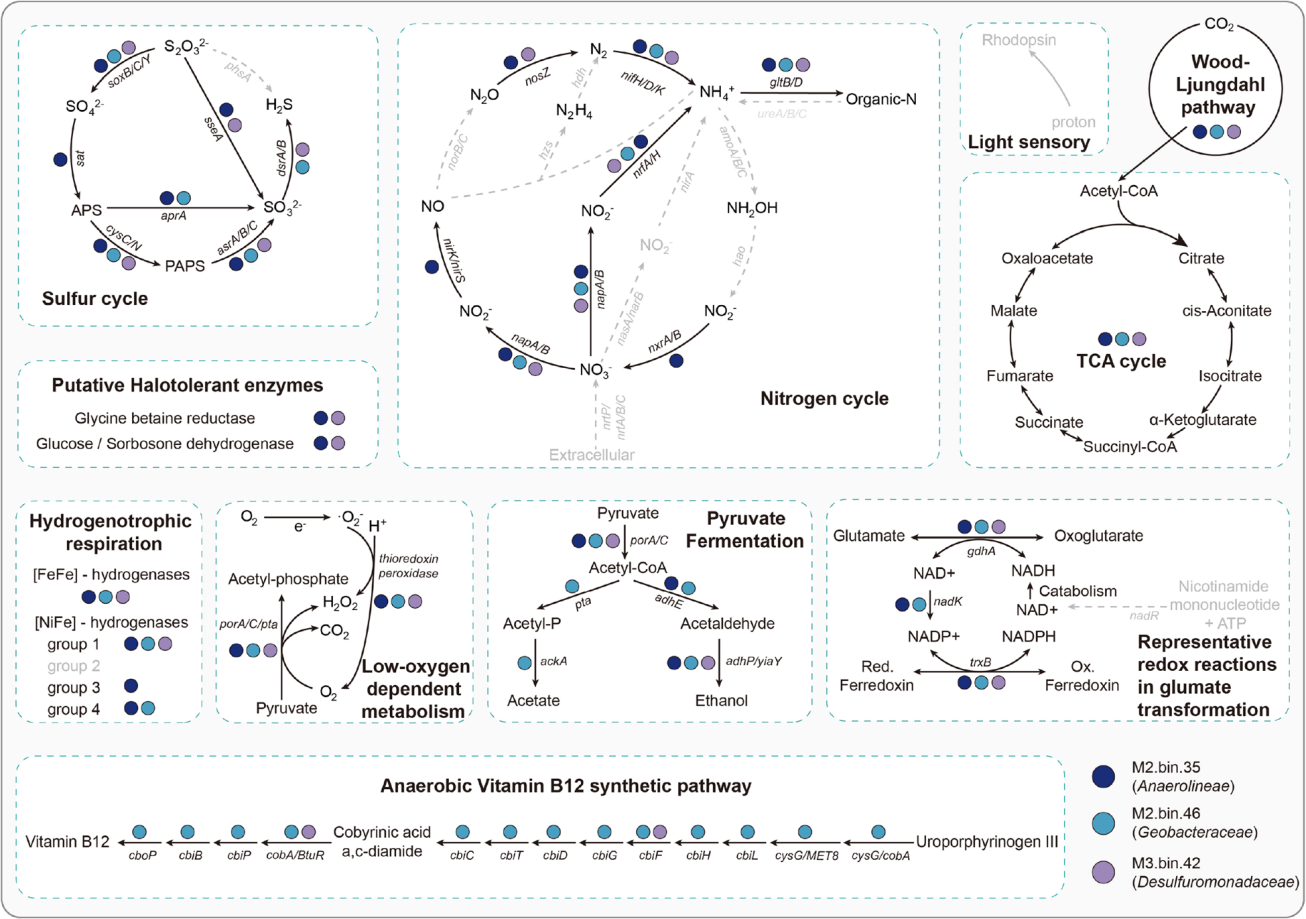
Metabolic characteristics of three diazotrophs projected on key pathways in mangrove sediments. Dark blue: metagenome-assembled genomes (MAGs) belonging to M2.bin.35 (*Anaerolineae*). Light blue: MAGs belonging to M2.bin.46 (*Geobacteraceae*). Light purple: MAGs belonging to M3.bin.42 (*Desulfuromonadaceae*). Black solid arrows indicate the genes/pathways that MAGs possessed, and gray dashed arrows indicate the missing genes/pathways. A detailed list of genes in these diazotrophs can be found in Additional file 4: Supplementary Data 2

De novo assembly and binning of metagenomic sequencing data from three depths of mangrove sediments allowed the reconstruction of 3 archaeal and 64 bacterial MAGs (completeness >50%, contamination <10%; Additional file 3: Supplementary Data 1). Given that metagenomic sequencing generated enormous data accompanied by tremendous undiscovered information, we inferred their potential physiological capabilities by annotating genes using the KAAS and TIFRFAM databases. Among all 67 MAGs, three MAGs possessed genes for nitrogen fixation (*nifH/D/K*), namely M2.bin.35, M2.bin.46, and M3.bin.42, which were affiliated to *Anaerolineae*, *Geobacteraceae*, and *Desulfuromonadaceae*, respectively (Fig. 5, Additional file 4: Supplementary Data 2). Interestingly, these three MAGs consistently contained genes associated with other nitrogen cycling processes, such as ammonia assimilation and the complete DNRA pathway (Fig. 5, Additional file 4: Supplementary Data 2). Additionally, M2.bin.35 had the genes involved with a nearly complete denitrification process except for converting NO to N_2_O (Fig. 5, Additional file 4: Supplementary Data 2). However, genes related to nitrate assimilation, assimilatory nitrate reduction, organic *N* decomposition, or nitrification were absent in these three diazotrophic MAGs (Fig. 5, Additional file 4: Supplementary Data 2).

Further functional annotations showed many potentials of these diazotrophic MAGs. From the perspective of energy metabolism, three MAGs contained genes involved in the complete or nearly complete carbon fixation pathways (such as Wood-Ljungdahl pathway) (Fig. 5, Additional file 4: Supplementary Data 2), which allowed them to convert inorganic carbon into organic molecules such as acetyl-CoA. Via TCA cycle, acetyl-CoA could be further utilized by these diazotrophs to generate energy for microbial metabolism (Fig. 5, Additional file 4: Supplementary Data 2). Together with the detection of fermentation genes encoding lactate dehydrogenase (*ldh*), pyruvate oxidoreductase (*porA/C*), and formate dehydrogenase (*fodG*) (Fig. 5, Additional file 4: Supplementary Data 2), our results suggested a mixotrophic lifestyle of these diazotrophs in mangrove sediments. From the perspective of adaptation strategy, diazotrophs from the middle and deep mangrove sediments contained functional genes for anaerobic respiration (*hyaB*, *hybC*) and its involved pathways (i.e., sulfur reduction), as well as anaerobic cobalamin biosynthesis (Fig. 5, Additional file 4: Supplementary Data 2). The adaptation of diazotrophs to the low-oxygen deep sediments was further supported by the genes related to pyruvate oxidoreductase (*porA/C*), thioredoxin peroxidase, cytochrome c oxidases (*coxA/B*), ccb_3_-type cytochrome c oxidases (*ccoN/O/P/Q*), and a cytochrome bd ubiquinol oxidase (*cydA*) (Fig. 5, Additional file 4: Supplementary Data 2). Furthermore, these diazotrophic MAGs were found to contain glycine betaine reductase and glucose/sorbosone dehydrogenase which can synthesize osmolytes [57, 58] to counteract osmotic stress in deep sediments with higher salinity. Together, these results indicated that the halotolerant diazotrophs in deep mangrove sediments were functionally versatile and facultative anaerobes.

## Discussion

Mangroves are considered as typical nitrogen-limited ecosystems [59]. Characterizing the biological nitrogen fixation and diazotrophic communities is, therefore, crucial to fully elucidate the nutrient status and ecological functions of mangrove ecosystems. In this study, we systematically examined the in-depth profile of NFR and diazotrophic communities across 10 depths of mangrove sediments. One of our prominent findings was that, relative to surficial sediments, diazotrophic communities are less diverse in deep sediments, where NFR is higher. Such depth-dependent variability of biological nitrogen fixation could be further supported by our metagenomic sequencing analysis, which revealed an elevated abundance of genes related to biological nitrogen fixation in deep sediments (Fig. 4). In line with the previous view that diazotrophs often accounted for a low percentage (0.95–2.50%) in a microbial community [60, 61], we found that only 3 out of 67 MAGs contained the nitrogen-fixation genes in our study. The functional annotations of diazotrophic MAGs provide genetic evidence for the functional versatility and adaptation strategies of diazotrophs under the low-oxygen and oligotrophic conditions of deep sediments. These results provide novel insights into the depth-dependent variability of NFR and diazotrophic communities and advance our understanding of the relationship between biological nitrogen fixation and nitrogen limitation in mangrove ecosystems.

Our in-depth survey of mangrove sediments revealed a clear divergence of diazotrophic community structure at the partitioning depth of 50 cm. Although the diversity of diazotrophic communities was lower in deep sediments than in surficial ones, the deep sediment-specific diazotrophs, including *Agrobacterium* and *Azotobacter*, potentially contributed to a higher NFR in deep sediments. There are two main reasons for this observation. First, due to the underground root distribution of *Sonneratia apetala* (about 40–60 cm) [62] and its root radial oxygen loss [63], we speculate that oxygen concentration decreased below 50 cm. It has been reported that metalloproteins of nitrogenase are extremely sensitive to oxygen, and the activities of MoFe protein and Fe protein in *Azotobacter* were sharply reduced when exposed to air [64]. As a result, the decreasing oxygen concentration with depth was thought to ensure the high activity of nitrogenase in deep mangrove sediments. Second, the lifestyle of diazotrophs was related to nitrogen fixation efficiency. Previous studies found that microaerophilic and anaerobic diazotrophs often exhibit a higher nitrogen fixation efficiency than aerobic diazotrophs [65]. In our study, the dominant diazotroph in the deep sediments was *Agrobacterium* sp., which has been reported to be a typical facultative anaerobe with the capability of anaerobic respiration in the presence of nitrate [66]. In line with this opinion, we confirmed the occurrence of genes related to low oxygen-dependent pathways in our diazotrophic MAGs (Fig. 5) and determined a facultatively anaerobic lifestyle of diazotrophs in deep mangrove sediments. Thus, the deep mangrove sediments with lower oxygen concentration could provide a suitable condition for microaerophilic/anaerobic diazotrophs to efficiently fix nitrogen [67], which is well consistent with higher NFR in deep sediments. Altogether, our results showed that changes in nitrogenase activity and shifts in the diazotrophic communities associated with mangrove sediments both contribute to the depth-dependent variability of biological nitrogen fixation observed under the fluctuating oxygen gradients typical of these environments.

Located at the transition between ocean and land, mangrove sediments experienced the tidal fluctuation day after day [68]. Probably due to the tidal flushing in surficial sediments and the bottom accumulation pattern of salinity in depth [69, 70], a continuous increase in salinity with the depth of mangrove sediments was observed in our study. Among the measured variables in our study, salinity was identified as the most important contributing factor for shaping the diazotrophic community structure in mangrove sediments, as revealed by our Mantel test and SEM results (Fig. 3, Additional file 2: Table S4). LEfSe analysis revealed that, *Methylomonas*, which was identified as less-salt-tolerant diazotroph [71], preferred to live in surficial sediments (Fig. 2b). However, in deep sediments with higher salinity, we observed that diazotrophic communities were dominated by *Azotobacter* and *Agrobacterium*, which belong to more-salt-tolerant diazotrophs [72, 73]. Such niche partition of diazotrophs across sediment depths may be closely tied to their salt-tolerant adaptation strategy. This is because diazotrophs thriving in higher-salinity sediments could apply the “low-salt-in” strategy to balance the osmotic potential of cytoplasm [74]. To support the adaptation strategy of diazotrophs to higher salinity, we did observe that diazotrophic MAGs contained genes encoding for glycine betaine reductase and glucose/sorbosone dehydrogenase (Fig. 5), which can synthesize osmolytes to balance the osmotic pressure created by higher-salinity habitats [57, 58]. Collectively, our study highlights the role of salinity in controlling the in-depth structure of diazotrophic communities and indicates the putative strategy of diazotrophs for salt tolerance in mangrove sediments.

Our current understanding of nitrogen-limited degrees in mangrove ecosystems is hitherto mainly limited to horizontal scales. For example, early evidence determined that the degree of nitrogen limitation in the fringe of mangrove forest is higher than that in the dwarf zone [24, 75]. Given that the dynamics of biological nitrogen fixation and its downstream processes in nitrogen cycling affected the degree of nitrogen limitation [76], we assumed that the mangrove nitrogen limitation status would also vary across vertical space, where variable nutrient (such as available nitrogen) dynamics and environmental gradients always occurred [77]. To support this assumption, our metagenomic sequencing analysis revealed that in deep mangrove sediments, the abundance of functional genes involved in biological nitrogen fixation (*nifH*) and ammonia assimilation (*gltB* and *gltD*) increased, whereas the abundance of functional genes related to denitrification (*nirK*, *norB,* and *norC*) deceased. These findings indicated that in the deeper depth, the amount of available nitrogen for mangrove growth tended to increase, and the loss of available nitrogen in the form of gas (such as N_2_O and N_2_) possibly decreased. Furthermore, due to the reduction of root density in deep mangrove sediments [62], the available nitrogen demand for mangrove growth decreased. Altogether, the dynamics of the supply and demand of available nitrogen across depths indicated a relieved nitrogen limitation in deep mangrove sediments.

## Conclusions

In summary, this study illustrates the depth-dependent variability of biological nitrogen fixation in mangrove sediments from the perspectives of both NFR and diazotrophic communities. The diversity of diazotrophic communities decreased with the depth of mangrove sediments, but the NFR and nitrogen fixation-related gene abundances increased. The salinity-driven structure of diazotrophic communities showed a clear divergence at the partitioning depth of 50 cm, as well as high abundances of *Azotobacter* and *Agrobacterium*, suggesting that *Azotobacter* and *Agrobacterium* may contribute greatly to the elevation of NFR in deep mangrove sediments. Accompanied by such an elevation, metagenomic sequencing analysis indicated that available nitrogen loss by denitrification pathway possibly decreased with depth. The depth-dependent variability of nitrogen fixation and its downstream processes in nitrogen cycling indicated the mitigation of nitrogen limitation in deep mangrove sediments. In addition, the MAGs of diazotrophs from deep mangrove sediments suggested their facultatively anaerobic and mixotrophic lifestyles. Overall, this study provides new insights into a comprehensive understanding of biological nitrogen fixation and its ecological functions in mangrove sediments.

## Supplementary Information

**Additional file 1:.** The detailed method for measuring nitrogen fixation rate by acetylene reduction assay.

**Additional file 2: Table S1.** A summary of reads information and microbial classification at different taxonomical levels. **Table S2.** Pearson’s correlations between the abundance of diazotrophic genera and the nitrogen fixation rate (NFR). **Table S3.** Relative abundances of diazotrophic communities at the genus level. **Table S4.** Mantel test for the correlations between diazotrophic community structure and sediment properties. **Fig. S1.** The location of the sampling habitats at the Qi’ao Island, Guangdong Province, China. **Fig. S2.** Relationships between the depth of mangrove sediments and the NFR (**a**), water content (**b**), salinity (**c**), Shannon index (**d**) and Chao1 index (**e**). NFR was represented as acetylene reduction (nmol g^-1^ h^-1^) in this research. Black lines and grey shaded areas represent linear regressions and 95% confidence intervals, respectively. R^2^ was obtained by linear regression analysis and *p* was obtained by Pearson’s correlation analysis. **Fig. S3.** The in-depth profile of physicochemical properties. The concentration of NH_4_^+^ (**a**), NO_2_^-^ (**b**), NO_3_^-^ (**c**), pH (**d**), water content (**e**), salinity (**f**), total Fe (**g**), available Fe (**h**) and Fe^3+^ (**i**) across 10 depths of mangrove sediments. Boxplots depict the 25-75% quantile range of the selected measurements, with the centerline depicting the median (50% quantile). Whiskers show the minimum and maximum values. **Fig. S4.** Bray-Curtis distance-based hierarchical clustering of diazotrophic communities across 10 depths of mangrove sediments. The color gradient represents sediment depth.

**Additional file 3: Supplementary Data 1**: Assembly statistics. The completeness, contamination and classification of 67 draft metagenome-assembled genomes.

**Additional file 4: Supplementary Data 2**: Summary of metabolic genes encoded within diazotrophic metagenome-assembled genomes (MAGs). The numbers represent the total number of metabolic genes identified in individual diazotrophic MAGs, and the red filled areas indicate the metabolic genes presented in these MAGs.

## Data Availability

The nucleotide sequences and metagenomic data of microbial communities in mangrove sediments were deposited in the SRA database under accession numbers PRJNA694572 and PRJNA698080. The authors declare that the primary data supporting the findings of this study are available within this article and in the additional files. Extra data supporting the findings of this study are available from the corresponding author upon request.

## Abbreviations

AB-DTPA: Ammonium bicarbonate - diethylenetriaminepentaacetic acid
CFI: Comparative fit index
CoA: Coenzyme A
DNRA: Dissimilatory nitrate reduction to ammonium
GFI: Goodness-of-fit index
LEfSe: Linear discriminant analysis effect size
ICP-OES: Inductively coupled plasma-optical emission spectrometer
KAAS: KEGG automatic annotation server
KEGG: Kyto Encyclopedia of Genes and Genomes
KO: KEGG orthology
MAG: Metagenome-assembled genome
NFR: Nitrogen fixation rate
ORFs: Open reading frames
OTUs: Operational taxonomic units
PCR: Polymerase chain reaction
PVC: Polyvinyl chloride
QIIME: Quantitative insights into microbial ecology
RMSEA: Root mean square error of approximation
rRNA: Ribosomal RNA
SEM: Structural equation model
sp.: Species
TCA: Tricarboxylic acid
STAMP: Statistical analysis of metagenomic profiles
TPM: Transcripts per million

## References

[1] Goldberg L, Lagomasino D, Thomas N, Fatoyinbo T (2020). Global declines in human-driven mangrove loss. Global Change Biol..

[2] Yu X, Yang X, Wu Y, Peng Y, Yang T, Xiao F, et al. *Sonneratia apetala* introduction alters methane cycling microbial communities and increases methane emissions in mangrove ecosystems. Soil Biol Biochem. 2020;144:107775. 10.1016/j.soilbio.2020.107775.

[3] Holguin G, Vazquez P, Bashan Y (2001). The role of sediment microorganisms in the productivity, conservation, and rehabilitation of mangrove ecosystems: an overview. Biol Fertility Soils..

[4] Inoue T, Kohzu A, Shimono A (2019). Tracking the route of atmospheric nitrogen to diazotrophs colonizing buried mangrove roots. Tree Physiol..

[5] Alfaro-Espinoza G, Ullrich MS. Bacterial N_2_-fixation in mangrove ecosystems: insights from a diazotroph-mangrove interaction. Front Microbiol. 2015;6(445). 10.3389/fmicb.2015.00445 PMC4426756 26029186

[6] Romero IC, Jacobson M, Fuhrman JA, Fogel M, Capone DG (2012). Long-term nitrogen and phosphorus fertilization effects on N_2_ fixation rates and *nifH* gene community patterns in mangrove sediments. Mar Ecol..

[7] Zhang Y, Dong J, Yang Z, Zhang S, Wang Y (2008). Phylogenetic diversity of nitrogen-fixing bacteria in mangrove sediments assessed by PCR-denaturing gradient gel electrophoresis. Arch Microbiol..

[8] Tang W, Cerdán-García E, Berthelot H, Polyviou D, Wang S, Baylay A, et al. New insights into the distributions of nitrogen fixation and diazotrophs revealed by high-resolution sensing and sampling methods. ISME J. 2020;14(10):2514–26. 10.1038/s41396-020-0703-6. 10.1038/s41396-020-0703-6 PMC7490393 32581316

[9] Kuypers MMM, Marchant HK, Kartal B (2018). The microbial nitrogen-cycling network. Nat Rev Microbiol..

[10] Inoue T, Shimono A, Akaji Y, Baba S, Takenaka A, Tuck CH (2019). Mangrove-diazotroph relationships at the root, tree and forest scales: diazotrophic communities create high soil nitrogenase activities in *Rhizophora stylosa* rhizospheres. Ann Bot..

[11] Jing H, Xia X, Liu H, Zhou Z, Wu C, Nagarajan S. Anthropogenic impact on diazotrophic diversity in the mangrove rhizosphere revealed by *nifH* pyrosequencing. Front Microbiol. 2015;6(1172). 10.3389/fmicb.2015.01172 PMC4612719 26539189

[12] Varon-Lopez M, Dias ACF, Fasanella CC, Durrer A, Melo IS, Kuramae EE, et al. Sulphur-oxidizing and sulphate-reducing communities in Brazilian mangrove sediments. Environ Microbiol. 2014;16(3):845–55. 10.1111/1462-2920.12237. 10.1111/1462-2920.12237 24033859

[13] Zhang Y, Yang Q, Ling J, Van Nostrand JD, Shi Z, Zhou J, et al. Diversity and structure of diazotrophic communities in mangrove rhizosphere, revealed by high-throughput sequencing. Front Microbiol. 2017;8(2032). 10.3389/fmicb.2017.02032 PMC5651520 29093705

[14] Lin X, Hetharua B, Lin L, Xu H, Zheng T, He Z, et al. Mangrove sediment microbiome: adaptive microbial assemblages and their routed biogeochemical processes in Yunxiao Mangrove National Nature Reserve. China. Microb Ecol. 2019;78(1):57–69. 10.1007/s00248-018-1261-6. 10.1007/s00248-018-1261-6 30284602

[15] Zhang M, Luo Y, La L, Lin X, Hetharua B, Zhao W (2018). Molecular and stable isotopic evidence for the occurrence of nitrite-dependent anaerobic methane-oxidizing bacteria in the mangrove sediment of Zhangjiang Estuary, China. Appl Microbiol Biotechnol..

[16] Wang Y, Li C, Kou Y, Wang J, Tu B, Li H, et al. Soil pH is a major driver of soil diazotrophic community assembly in Qinghai-Tibet alpine meadows. Soil Biol Biochem. 2017;115:547–55. 10.1016/j.soilbio.2017.09.024.

[17] Flores-Mireles AL, Winans SC, Holguin G (2007). Molecular characterization of diazotrophic and denitrifying bacteria associated with mangrove roots. Appl Environ Microbiol..

[18] Zhang G, Bai J, Tebbe CC, Zhao Q, Jia J, Wang W, et al. Salinity controls soil microbial community structure and function in coastal estuarine wetlands. Environ Microbiol. 2021;23(2):1020–37. 10.1111/1462-2920.15281. 10.1111/1462-2920.15281 33073448

[19] Heděnec P, Rui J, Lin Q, Yao M, Li J, Li H, et al. Functional and phylogenetic response of soil prokaryotic community under an artificial moisture gradient. Appl Soil Ecol. 2018;124:372–8. 10.1016/j.apsoil.2017.12.009.

[20] Chowdhury N, Marschner P, Burns R (2011). Response of microbial activity and community structure to decreasing soil osmotic and matric potential. Plant Soil..

[21] Averill C, Rousk J, Hawkes C (2015). Microbial-mediated redistribution of ecosystem nitrogen cycling can delay progressive nitrogen limitation. Biogeochemistry..

[22] Moreau D, Bardgett RD, Finlay RD, Jones DL, Philippot L (2019). A plant perspective on nitrogen cycling in the rhizosphere. Funct Ecol..

[23] Russell DG, Warry FY, Cook PLM (2016). The balance between nitrogen fixation and denitrification on vegetated and non-vegetated intertidal sediments. Limnol Oceanogr..

[24] Reis CRG, Nardoto GB, Oliveira RS (2017). Global overview on nitrogen dynamics in mangroves and consequences of increasing nitrogen availability for these systems. Plant Soil..

[25] Li S, Meng X, Ge Z, Zhang L (2015). Vulnerability assessment of the coastal mangrove ecosystems in Guangxi, China, to sea-level rise. Reg Environ Change..

[26] Yu C, Feng J, Liu K, Wang G, Zhu Y, Chen H, et al. Changes of ecosystem carbon stock following the plantation of exotic mangrove *Sonneratia apetala* in Qi’ao Island. China. Sci Total Environ. 2020;717:137142. 10.1016/j.scitotenv.2020.137142. 10.1016/j.scitotenv.2020.137142 32070894

[27] Han L, Wang Q, Shen J, Di HJ, Wang J, Wei W, et al. Multiple factors drive the abundance and diversity of the diazotrophic community in typical farmland soils of China. FEMS Microbiol Ecol. 2019;95(8). 10.1093/femsec/fiz113 31295349

[28] Das S, De TK (2018). Microbial assay of N_2_ fixation rate, a simple alternate for acetylene reduction assay. MethodsX..

[29] Soltanpour PN, Workman S (1979). Modification of the NH_4_HCO_3_-DTPA soil test to omit carbon black. Commun Soil Sci Plant Anal..

[30] Cai C, Leu AO, Xie G, Guo J, Feng Y, Zhao J (2018). A methanotrophic archaeon couples anaerobic oxidation of methane to Fe(III) reduction. ISME J..

[31] Zhou J, Bruns MA, Tiedje JM (1996). DNA recovery from soils of diverse composition. Appl Environ Microbiol..

[32] Poly F, Monrozier LJ, Bally R (2001). Improvement in the RFLP procedure for studying the diversity of *nifH* genes in communities of nitrogen fixers in soil. Res Microbiol..

[33] Martin M (2011). Cutadapt removes adapter sequences from high-throughput sequencing reads. EMBnet J..

[34] Magoč T, Salzberg SL (2011). FLASH: fast length adjustment of short reads to improve genome assemblies. Bioinformatics..

[35] Edgar RC, Haas BJ, Clemente JC, Quince C, Knight R (2011). UCHIME improves sensitivity and speed of chimera detection. Bioinformatics..

[36] Wang Q, Quensen JF, Fish JA, Lee TK, Sun Y, Tiedje JM (2013). Ecological patterns of *nifH* genes in four terrestrial climatic zones explored with targeted metagenomics using FrameBot, a new Informatics Tool. mBio.

[37] Feng J, Penton CR, He Z, Nostrand JDV, Yuan MM (2019). Wu L, et al. Long-term warming in Alaska enlarges the diazotrophic community in deep soils. mBio..

[38] Edgar RC (2013). UPARSE: highly accurate OTU sequences from microbial amplicon reads. Nat Methods..

[39] Tu Q, Zhou X, He Z, Xue K, Wu L, Reich P, et al. The diversity and co-occurrence patterns of N_2_-fixing communities in a CO_2_-enriched grassland ecosystem. Microb Ecol. 2016;71(3):604–15. 10.1007/s00248-015-0659-7. 10.1007/s00248-015-0659-7 26280746

[40] Fish J, Chai B, Wang Q, Sun Y, Brown CT, Tiedje J, et al. FunGene: the functional gene pipeline and repository. Front Microbiol. 2013;4(291). 10.3389/fmicb.2013.00291 PMC3787254 24101916

[41] Gilbert JA, Field D, Swift P, Thomas S, Cummings D, Temperton B, et al. The taxonomic and functional diversity of microbes at a temperate coastal site: a ‘multi-omic’ study of seasonal and diel temporal variation. Plos One. 2010;5(11). 10.1371/journal.pone.0015545 PMC2993967 21124740

[42] Li D, Liu C, Luo R, Sadakane K, Lam T (2015). MEGAHIT: an ultra-fast single-node solution for large and complex metagenomics assembly via succinct de Bruijn graph. Bioinformatics..

[43] Fu L, Niu B, Zhu Z, Wu S, Li W (2012). CD-HIT: accelerated for clustering the next-generation sequencing data. Bioinformatics..

[44] Seyler LM, Trembath-Reichert E, Tully BJ, Huber JA (2021). Time-series transcriptomics from cold, oxic subseafloor crustal fluids reveals a motile, mixotrophic microbial community. ISME J..

[45] Uritskiy GV, DiRuggiero J, Taylor J (2018). MetaWRAP-a flexible pipeline for genome-resolved metagenomic data analysis. Microbiome..

[46] Kang DD, Li F, Kirton E, Thomas A, Egan R, An H, et al. MetaBAT 2: an adaptive binning algorithm for robust and efficient genome reconstruction from metagenome assemblies. PeerJ. 2019;7:e7359. 10.7717/peerj.7359. 10.7717/peerj.7359 PMC6662567 31388474

[47] Wu Y, Simmons BA, Singer SW (2015). MaxBin 2.0: an automated binning algorithm to recover genomes from multiple metagenomic datasets. Bioinformatics..

[48] Patro R, Duggal G, Love MI, Irizarry RA, Kingsford C (2017). Salmon provides fast and bias-aware quantification of transcript expression. Nat Methods..

[49] Parks DH, Chuvochina M, Waite DW, Rinke C, Skarshewski A, Chaumeil P-A, et al. A standardized bacterial taxonomy based on genome phylogeny substantially revises the tree of life. Nat Biotechnol. 2018;36(10):996–1004. 10.1038/nbt.4229. 10.1038/nbt.4229 30148503

[50] Moriya Y, Itoh M, Okuda S, Yoshizawa AC, Kanehisa M (2007). KAAS: an automatic genome annotation and pathway reconstruction server. Nucleic Acids Res..

[51] Dombrowski N, Teske AP, Baker BJ (2018). Expansive microbial metabolic versatility and biodiversity in dynamic Guaymas Basin hydrothermal sediments. Nat Commun..

[52] Parks DH, Beiko RG (2010). Identifying biologically relevant differences between metagenomic communities. Bioinformatics..

[53] Eyice Ö, Namura M, Chen Y, Mead A, Samavedam S, Schäfer H (2015). SIP metagenomics identifies uncultivated Methylophilaceae as dimethylsulphide degrading bacteria in soil and lake sediment. ISME J..

[54] Chen L, Jiang Y, Liang C, Luo Y, Xu Q, Han C, et al. Competitive interaction with keystone taxa induced negative priming under biochar amendments. Microbiome. 2019;7(1):77. 10.1186/s40168-019-0693-7. 10.1186/s40168-019-0693-7 PMC6526607 31109381

[55] Prayitno J, Rolfe B (2010). Characterization of endophytic diazotroph bacteria isolated from rice. HAYATI J Biosci..

[56] Inomura K, Bragg J, Riemann L, Follows MJ. A quantitative model of nitrogen fixation in the presence of ammonium. Plos One. 2018;13(11). 10.1371/journal.pone.0208282 PMC6264846 30496286

[57] Figueroa-Soto CG, Valenzuela-Soto EM (2018). Glycine betaine rather than acting only as an osmolyte also plays a role as regulator in cellular metabolism. Biochimie..

[58] Ahmed V, Verma MK, Gupta S, Mandhan V, Chauhan NS. Metagenomic profiling of soil microbes to mine salt stress tolerance genes. Front Microbiol. 2018;9(159). 10.3389/fmicb.2018.00159 PMC5809485 29472909

[59] Liao S, Wang Y, Liu H, Fan G, Sahu SK (2020). Jin T, et al. Deciphering the microbial taxonomy and functionality of two diverse mangrove ecosystems and their potential abilities to produce bioactive compounds. mSystems..

[60] Hilton JA, Satinsky BM, Doherty M, Zielinski B, Zehr JP (2015). Metatranscriptomics of N_2_-fixing cyanobacteria in the Amazon River plume. ISME J..

[61] Delmont TO, Karlusich JJP, Veseli I, Fuessel J, Eren AM, Foster RA, et al. Heterotrophic bacterial diazotrophs are more abundant than their cyanobacterial counterparts in metagenomes covering most of the sunlit ocean. bioRxiv. 2021:2021.2003.2024.436778.

[62] Du Q, Li L (2018). Temporal-spatial distribution features in the root system of individual *Sonneratia apetala* and *Avicennia marina* plants. Acta Ecologica Sinica..

[63] Yin Y, Yan Z (2020). Variations of soil bacterial diversity and metabolic function with tidal flat elevation gradient in an artificial mangrove wetland. Sci Total Environ..

[64] Robson RL, Postgate JR (1980). Oxygen and hydrogen in biological nitrogen fixation. Annu Rev Microbiol..

[65] Smercina DN, Evans SE, Friesen ML, Tiemann LK, Cann I (2019). To fix or not to fix: controls on free-living nitrogen fixation in the rhizosphere. Appl Environ Microbiol..

[66] Lee BD, Ellis JT, Dodwell A, Eisenhauer EER, Saunders DL, Lee MH (2018). Iodate and nitrate transformation by *Agrobacterium*/*Rhizobium* related strain DVZ35 isolated from contaminated Hanford groundwater. J Hazard Mater..

[67] Jean MRN, Gonzalez-Rizzo S, Gauffre-Autelin P, Lengger SK, Schouten S, Gros O. Two new *Beggiatoa* species inhabiting marine mangrove sediments in the Caribbean. PloS one. 2015;10(2):e0117832–2. 10.1371/journal.pone.0117832. 10.1371/journal.pone.0117832 PMC4331518 25689402

[68] Collins DS, Avdis A, Allison PA, Johnson HD, Hill J, Piggott MD, et al. Tidal dynamics and mangrove carbon sequestration during the Oligo-Miocene in the South China Sea. Nat Commun. 2017;8(1):15698. 10.1038/ncomms15698. 10.1038/ncomms15698 PMC5481738 28643789

[69] Feng X, Xu S, Li J, Yang Y, Chen Q, Lyu H, et al. Molecular adaptation to salinity fluctuation in tropical intertidal environments of a mangrove tree *Sonneratia alba*. BMC Plant Biol. 2020;20(1):178. 10.1186/s12870-020-02395-3. 10.1186/s12870-020-02395-3 PMC7178616 32321423

[70] Fu Z, Wang P, Sun J, Lu Z, Yang H, Liu J, et al. Composition, seasonal variation, and salinization characteristics of soil salinity in the Chenier Island of the Yellow River Delta. Glob Ecol Conserv. 2020;24:e01318. 10.1016/j.gecco.2020.e01318.

[71] Zhang Y, Chen J, Wen L, Tang Y, Zhao H (2016). Effects of salinity on simultaneous reduction of perchlorate and nitrate in a methane-based membrane biofilm reactor. Environ Sci Pollut R..

[72] Yan J, Li Y, Yan H, Chen WF, Zhang X, Wang ET, et al. *Agrobacterium salinitolerans* sp. nov., a saline-alkaline-tolerant bacterium isolated from root nodule of *Sesbania cannabina*. Int J Syst Evol Microbiol. 2017;67(6):1906–11. 10.1099/ijsem.0.001885. 10.1099/ijsem.0.001885 28629499

[73] Van Oosten MJ, Di Stasio E, Cirillo V, Silletti S, Ventorino V, Pepe O (2018). Root inoculation with *Azotobacter chroococcum* 76A enhances tomato plants adaptation to salt stress under low N conditions. BMC Plant Biol..

[74] Oren A. Life at high salt concentrations, intracellular KCl concentrations, and acidic proteomes. Front Microbiol. 2013;4(315). 10.3389/fmicb.2013.00315 PMC3817357 24204364

[75] Feller IC, McKee KL, Whigham DF, O'Neill JP (2003). Nitrogen vs. phosphorus limitation across an ecotonal gradient in a mangrove forest. Biogeochemistry..

[76] Meiyappan P, Jain AK, House JI (2015). Increased influence of nitrogen limitation on CO_2_ emissions from future land use and land use change. Global Biogeochem Cy..

[77] Kaiser D, Kowalski N, Böttcher ME, Yan B, Unger D (2015). Benthic nutrient fluxes from mangrove sediments of an anthropogenically impacted estuary in southern China. J Mar Sci Eng..

