# Supplementary material for: Depth-dependent variability of biological nitrogen fixation and diazotrophic communities in mangrove sediments
Source: Microbiome. 2021 Oct 27;9:212. doi: 10.1186/s40168-021-01164-0 (PMC8549172; doi:10.1186/s40168-021-01164-0)
Supplement: Supplementary file 2 — Additional file 1:. The detailed method for measuring nitrogen fixation rate by acetylene reduction assay. [file 40168_2021_1164_MOESM2_ESM.pdf]

Additional file 1: The detailed method for measuring nitrogen fixation rate by acetylene reduction assay.

Acetylene reduction assay

Acetylene reduction assay is used to determine sediment nitrogenase activity (Han et al, 2019). Fresh sediment (10g) was weighed and put into 100 mL serum vial. The vials were sealed with rubber stoppers and 10% of the headspace was replaced with pure and fresh acetylene (C_2H_2) before they were incubated in dark at 25 °C. After incubation for 48 h, 200 μL headspace gas was taken out to measure the concentration of ethylene (C_2H_4) by Agilent gas chromatograph (HP7890B, Agilent, USA) equipped with a flame ionization detector and a HP-PLOT MoleSieve5A capillary column (30.0 m × 530 μm × 50 μm) (Agilent, USA). The chromatograms were used to integrate the areas of C_2H_4 to estimate C_2H_4 productions (Das and De, 2018). As the C_2H_4 productions from the chromatograms only represent the molar mass of C_2H_4 ($M_{C_2H_4}$, nmol) of the 200 μL headspace gas, the total C_2H_4 concentrations should multiply 500 (that is the total volume (100 mL) divide the measured volume (200 μL). In short, the total C_2H_4 productions = $M_{C_2H_4} \times 500$ (nmol).

Nitrogen fixation rate

Nitrogen fixation rate (NFR) was represented as acetylene reduction (nmol C_2H_4 $g^{-1}h^{-1}$) in this research:

$$NFR = \frac{\text{Total } \mathrm{C}_2H_4 \text{ production}}{\text{Incubation time } \times \text{mass of sediment}} = \frac{M_{C_2H_4} \times 500 \ (nmol)}{48 \ (h) \ \times 10 \ (g)}$$

$$= \frac{25}{24} M_{C_2H_4} \ (noml \ \mathrm{C}_2H_4 \ g^{-1}h^{-1})$$

References:

Han L, Wang Q, Shen J, Di HJ, Wang J, Wei W, et al. Multiple factors drive the abundance and diversity of the diazotrophic community in typical farmland soils of China. FEMS Microbiol Ecol. 2019;95(8).

Das S, De TK. Microbial assay of N2 fixation rate, a simple alternate for acetylene reduction assay. MethodsX. 2018;5:909-914.
