# Supplementary material for: Depth-dependent variability of biological nitrogen fixation and diazotrophic communities in mangrove sediments
Source: Microbiome. 2021 Oct 27;9:212. doi: 10.1186/s40168-021-01164-0 (PMC8549172; doi:10.1186/s40168-021-01164-0)
Supplement: Supplementary file 3 — Additional file 2: Table S1. A summary of reads information and microbial classification at different taxonomical levels. Table S2. Pearson’s correlations between the abundance of diazotrophic genera and the nitrogen fixation rate (NFR). Table S3. Relative abundances of diazotrophic communities at the genus level. Table S4. Mantel test for the correlations between diazotrophic community structure and sediment properties. Fig. S1. The location of the sampling habitats at the Qi’ao Island, Guangdong Province, China. Fig. S2. Relationships between the depth of mangrove sediments and the NFR (a), water content (b), salinity (c), Shannon index (d) and Chao1 index (e). NFR was represented as acetylene reduction (nmol g-1 h-1) in this research. Black lines and grey shaded areas represent linear regressions and 95% confidence intervals, respectively. R2 was obtained by linear regression analysis and p was obtained by Pearson’s correlation analysis. Fig. S3. The in-depth profile of physicochemical properties. The concentration of NH4+ (a), NO2- (b), NO3- (c), pH (d), water content (e), salinity (f), total Fe (g), available Fe (h) and Fe3+ (i) across 10 depths of mangrove sediments. Boxplots depict the 25-75% quantile range of the selected measurements, with the centerline depicting the median (50% quantile). Whiskers show the minimum and maximum values. Fig. S4. Bray-Curtis distance-based hierarchical clustering of diazotrophic communities across 10 depths of mangrove sediments. The color gradient represents sediment depth. [file 40168_2021_1164_MOESM3_ESM.pdf]

A. Supplementary tables

Table S1. A summary of reads information and microbial classification at different taxonomic levels.

Table S2. Pearson's correlations between the abundance of diazotrophic genera and the nitrogen fixation rate (NFR).

Table S3. Relative abundances of diazotrophic communities at the genus level.

Table S4. Mantel test for the correlations between diazotrophic community structure and sediment properties.

B. Supplementary figures

Fig. S1. The location of the sampling habitats at the Qi'ao Island, Guangdong Province, China.

Fig. S2. Relationships between the depth of mangrove sediments and the NFR (**a**), water content (**b**), salinity (**c**), Shannon index (**d**) and Chao1 index (**e**). NFR was represented as acetylene reduction (nmol g^{-1} h^{-1}) in this research. Black lines and grey shaded areas represent linear regressions and 95% confidence intervals, respectively. R^2 was obtained by linear regression analysis and *p* was obtained by Pearson's correlation analysis.

Fig. S3. The in-depth profile of physicochemical properties. The concentration of NH_4^+ (**a**), NO_2^- (**b**), NO_3^- (**c**), pH (**d**), water content (**e**), salinity (**f**), total Fe (**g**), available Fe (**h**) and Fe^{3+} (**i**) across 10 depths of mangrove sediments. Boxplots depict the 25-75% quantile range of the selected measurements, with the centerline depicting the median (50% quantile). Whiskers show the minimum and maximum values.

Fig. S4. Bray-Curtis distance-based hierarchical clustering of diazotrophic communities across 10 depths of mangrove sediments. The color gradient represents sediment depth.

A. Supplementary tables

Table S1. A summary of reads information and microbial classification at different taxonomical levels.

Samples	Reads	OTUs	Phylum	Class	Order	Family	Genus	Species
S1-0-10cm	86195	718	5	9	22	34	49	90
S2-0-10cm	70481	758	5	10	22	34	52	94
S3-0-10cm	80416	772	6	10	22	35	51	94
S4-10-20cm	66846	820	5	10	24	37	56	101
S5-10-20cm	100501	789	5	10	23	36	54	96
S6-10-20cm	81996	736	6	11	23	36	54	98
S7-20-30cm	68499	779	6	10	21	35	52	97
S8-20-30cm	64514	787	5	10	22	36	53	97
S9-20-30cm	87197	776	5	10	23	37	56	98
S10-30-40cm	83713	783	5	10	23	37	54	96
S11-30-40cm	74631	690	3	8	21	31	46	85
S12-30-40cm	66249	805	5	10	24	38	53	94
S13-40-50cm	67937	854	6	11	24	37	55	100
S14-40-50cm	73389	747	4	8	21	34	52	91
S15-40-50cm	91327	843	5	10	23	37	54	97
S16-50-60cm	73672	863	6	11	25	39	56	99
S17-50-60cm	54512	748	6	11	24	37	52	92
S18-50-60cm	58440	816	4	9	23	37	55	96
S19-60-70cm	89734	789	6	11	23	37	54	96
S20-60-70cm	86355	625	6	11	25	37	53	86
S21-60-70cm	74963	664	5	10	23	35	52	89
S22-70-80cm	74963	725	6	10	23	35	50	91

S23-70-80cm	104660	719	6	11	24	36	51	90
S24-70-80cm	100077	627	4	8	22	31	46	79
S25-80-90cm	54074	696	5	10	23	35	53	92
S26-80-90cm	65338	758	5	10	23	35	50	92
S27-80-90cm	71914	579	6	11	24	36	48	77
S28-90-100cm	57216	777	6	11	25	38	55	96
S29-90-100cm	60295	654	5	10	23	36	50	86
S30-90-100cm	60282	761	5	11	24	37	55	91
Total	2253352	974	6	11	25	39	58	103

Table S2. Pearson's correlations between the abundance of diazotrophic genera and the nitrogen fixation rate (NFR).

Genus	*Bradyrhizobium*	*Geobacter*	*Azotobacter*	*Halorhodospira*	*unidentified*	*Desulfovibrio*
r-value	-0.326	-0.277	0.693*	-0.507	0.540	-0.750*
p-value	0.358	0.439	0.026	0.135	0.107	0.012
Genus	*Dechloromonas*	*Agrobacterium*	*Anaeromyxobacter*	*Marichromatium*	*Pelobacter*	*Desulfobulbus*
r-value	0.003	0.853**	-0.726*	-0.830**	0.430	0.161
p-value	0.993	0.002	0.017	0.003	0.215	0.657
Genus	*Pseudomonas*	*Rubrivivax*	*Azohydromonas*	*Pseudodesulfovibrio*	*Methylobacterium*	*Desulfatibacillum*
r-value	-0.756*	-0.711*	-0.216	-0.433	-0.677*	0.478
p-value	0.010	0.021	0.549	0.211	0.031	0.162
Genus	*Sinorhizobium*	*Azoarcus*	*Zoogloea*	*Pelomonas*	*Ideonella*	*Azospirillum*
r-value	-0.186	-0.464	0.563	-0.581	0.444	-0.674*
p-value	0.607	0.177	0.090	0.078	0.198	0.033
Genus	*Dehalococcoides*	*Geoalkalibacter*	*Ectothiorhodospira*	*Methylomonas*	*Desulfuromonas*	*Dickeya*
r-value	0.122	-0.426	-0.282	-0.638*	0.450	-0.648*
p-value	0.738	0.219	0.431	0.047	0.192	0.043
Genus	*Sideroxydans*	*Thiocapsa*	*Desulfarculus*	*Sulfuricurvum*	*Chlorobaculum*	*Azomonas*
r-value	-0.533	-0.729*	-0.567	0.163	0.323	-0.345
p-value	0.112	0.017	0.087	0.652	0.362	0.329
Genus	*Methylocystis*	*Klebsiella*	*Methanoregula*	*Azorhizobium*	*Rhodobacter*	*Xanthobacter*
r-value	-0.772**	0.325	0.246	-0.168	-0.207	-0.484
p-value	0.009	0.360	0.493	0.642	0.567	0.156
Genus	*Pelosinus*	*Allochromatium*	*Heliobacterium*	*Desulfurivibrio*	*Methanothrix*	*Nitratireductor*
r-value	-0.618	0.408	-0.289	-0.021	0.148	-0.116

p-value	0.057	0.241	0.418	0.953	0.683	0.749
Genus	*Methanocella*	*Thioalkalispira*	*Magnetospirillum*	*Azospira*	*Thiorhodococcus*	*Vibrio*
r-value	-0.186	-0.373	-0.546	0.538	-0.213	0.096
p-value	0.607	0.288	0.103	0.108	0.554	0.193
Genus	*Leptothrix*	*Candidatus_Accumulibacter*	*Paraburkholderia*	*Rhodopseudomonas*	*Methylococcus*	
r-value	-0.429	-0.101	-0.383	-0.482	-0.620	
p-value	0.216	0.780	0.274	0.158	0.056	

Note: *: $p < 0.05$ means a significant correlation, **: $p < 0.01$ is very significant.

Table S3. Relative abundances of diazotrophic communities at the genus level.

Genus	*Bradyrhizobium*	*Geobacter*	*Azotobacter*	*Halorhodospira*	*unidentified*	*Desulfovibrio*
Average relative abundance (%)	15.18 ± 5.51	14.74 ± 4.87	11.92 ± 13.9	11.12 ± 4.46	8.59 ±5.20	5.19 ± 3.51
Genus	*Dechloromonas*	*Agrobacterium*	*Anaeromyxobacter*	*Marichromatium*	*Pelobacter*	*Desulfobulbus*
Average relative abundance (%)	3.51 ± 3.87	3.28 ± 5.19	2.88 ± 3.12	2.81 ± 1.21	2.68 ± 1.67	2.64 ± 1.69
Genus	*Pseudomonas*	*Rubrivivax*	*Azohydromonas*	*Pseudodesulfovibrio*	*Methylobacterium*	*Desulfatibacillum*
Average relative abundance (%)	1.70 ± 1.09	1.64 ± 1.39	1.38 ± 2.43	1.32 ± 0.86	1.01 ± 0.81	0.91 ± 2.30
Genus	*Sinorhizobium*	*Azoarcus*	*Zoogloea*	*Pelomonas*	*Ideonella*	*Azospirillum*
Average relative abundance (%)	0.70 ± 0.91	0.58 ± 0.71	0.52 ± 0.50	0.51 ± 0.94	0.48 ± 0.38	0.45 ± 0.44
Genus	*Dehalococcoides*	*Geoalkalibacter*	*Ectothiorhodospira*	*Methylomonas*	*Desulfuromonas*	*Dickeya*
Average relative abundance (%)	0.45 ± 1.32	0.44 ± 0.59	0.40 ± 0.33	0.33 ± 0.41	0.28 ± 0.70	0.25 ± 0.22
Genus	*Sideroxydans*	*Thiocapsa*	*Desulfarculus*	*Sulfuricurvum*	*Chlorobaculum*	*Azomonas*
Average relative abundance (%)	0.18 ± 0.23	0.18 ± 0.12	0.14 ± 0.12	0.14 ± 0.19	0.12 ± 0.39	0.12 ± 0.33
Genus	*Methylocystis*	*Klebsiella*	*Methanoregula*	*Azorhizobium*	*Rhodobacter*	*Xanthobacter*

Average relative abundance (%)	0.11 ± 0.11	0.09 ± 0.20	0.08 ± 0.27	0.08 ± 0.13	0.08 ± 0.06	0.08 ± 0.06
Genus	*Pelosinus*	*Allochromatium*	*Heliobacterium*	*Desulfurivibrio*	*Methanothrix*	*Nitratireductor*
Average relative abundance (%)	0.08 ± 0.09	0.07 ± 0.08	0.06 ± 0.09	0.6 ± 0.08	0.05 ± 0.05	0.05 ± 0.09
Genus	*Methanocella*	*Thioalkalispira*	*Magnetospirillum*	*Azospira*	*Thiorhodococcus*	*Vibrio*
Average relative abundance (%)	0.05 ± 0.06	0.05 ± 0.14	0.04 ± 0.05	0.03 ± 0.04	0.03 ± 0.05	0.03 ± 0.04
Genus	*Leptothrix*	*Candidatus_ Accumulibacter*	*Paraburkholderia*	*Rhodopseudomonas*	*Methylococcus*	
Average relative abundance (%)	0.02 ± 0.04	0.02 ± 0.05	0.02 ± 0.14	0.02 ± 0.03	0.02 ± 0.02	

Note: Data are shown as means ± SD.

Table S4. Mantel test for the correlations between diazotrophic community structure and sediment properties.

Correlation between diazotrophic community structure and:	Mantel r	*p*
Salinity	0.477**	**< 0.01**
pH	0.240**	**< 0.01**
Fe^{3+}	0.237**	**< 0.01**
Water content	0.209**	**< 0.01**
NH_4^+	0.192*	**< 0.05**
NO_3^-	0.075	≥ 0.05
NO_2^-	0.010	≥ 0.05
Total Fe	0.002	≥ 0.05
Available Fe	-0.06	≥ 0.05

Note: Bold value indicates $p < 0.05$. (*: $p < 0.05$; **: $p < 0.01$)

B. Supplementary figures

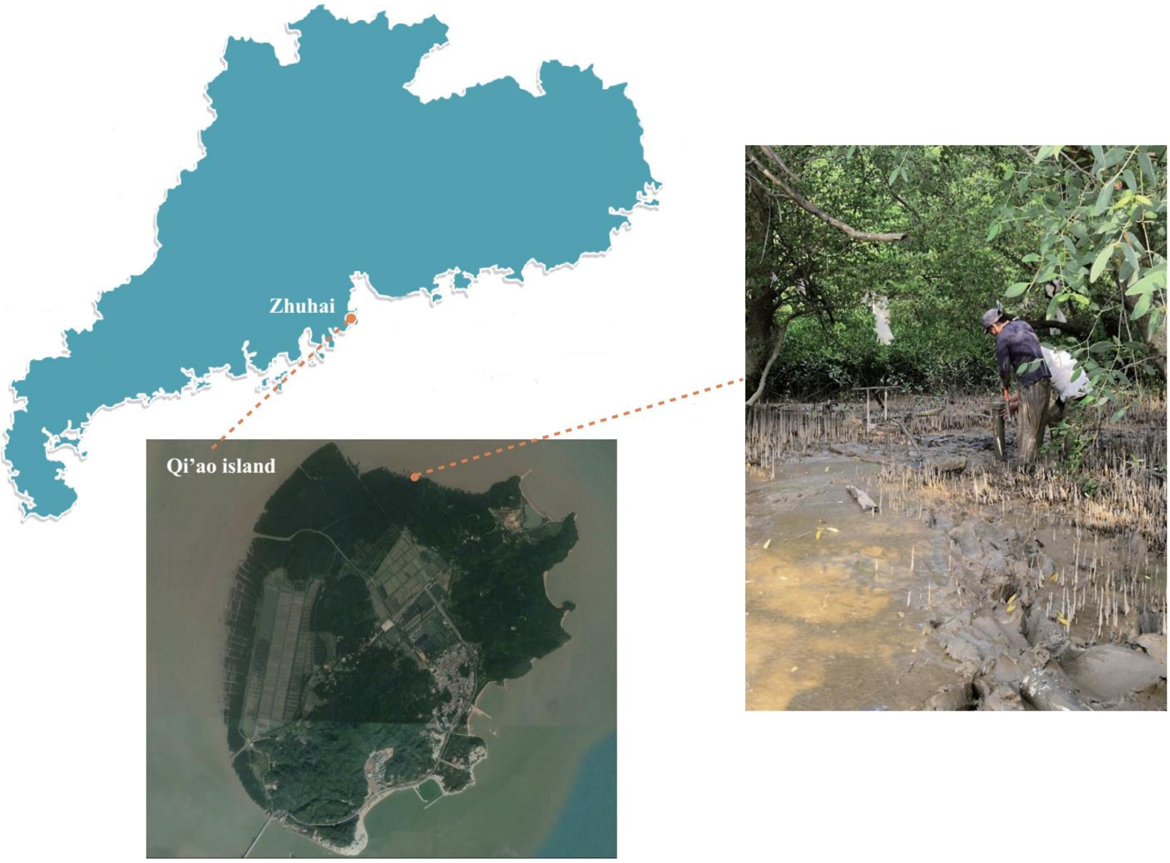

Fig. S1. The location of the sampling habitats at the Qi’ao Island, Guangdong Province, China.

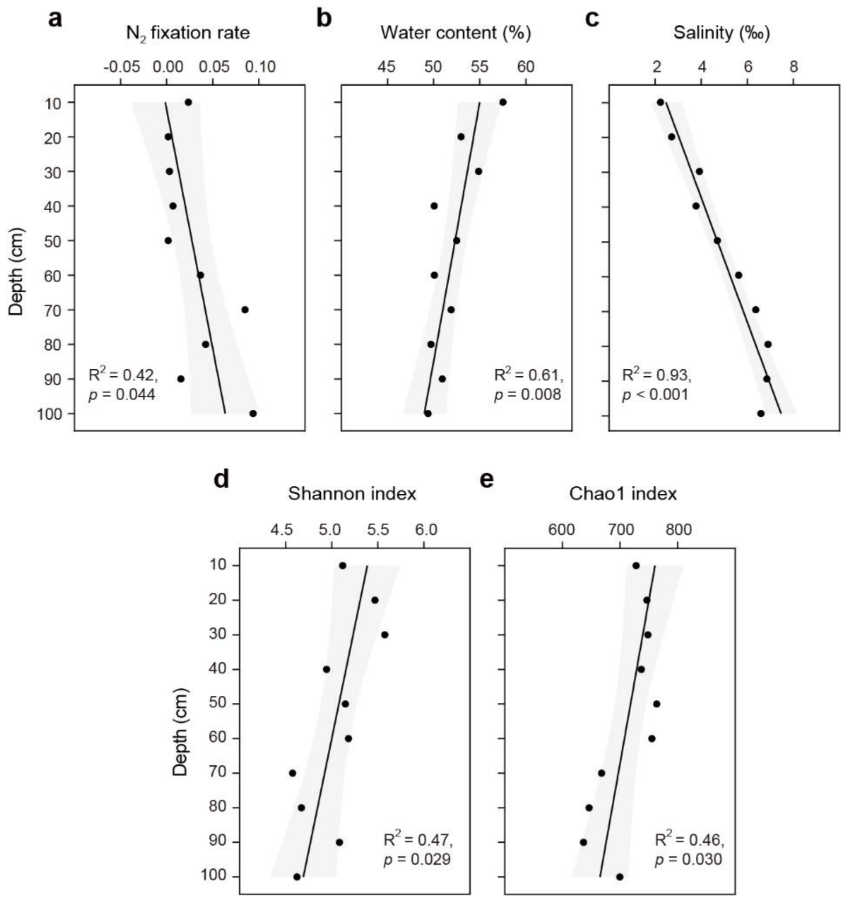

Fig. S2. Relationships between the depth of mangrove sediments and the NFR (**a**), water content (**b**), salinity (**c**), Shannon index (**d**) and Chao1 index (**e**). NFR was represented as acetylene reduction (nmol g^{-1} h^{-1}) in this research. Black lines and grey shaded areas represent linear regressions and 95% confidence intervals, respectively. R^2 was obtained by linear regression analysis and *p* was obtained by Pearson's correlation analysis.

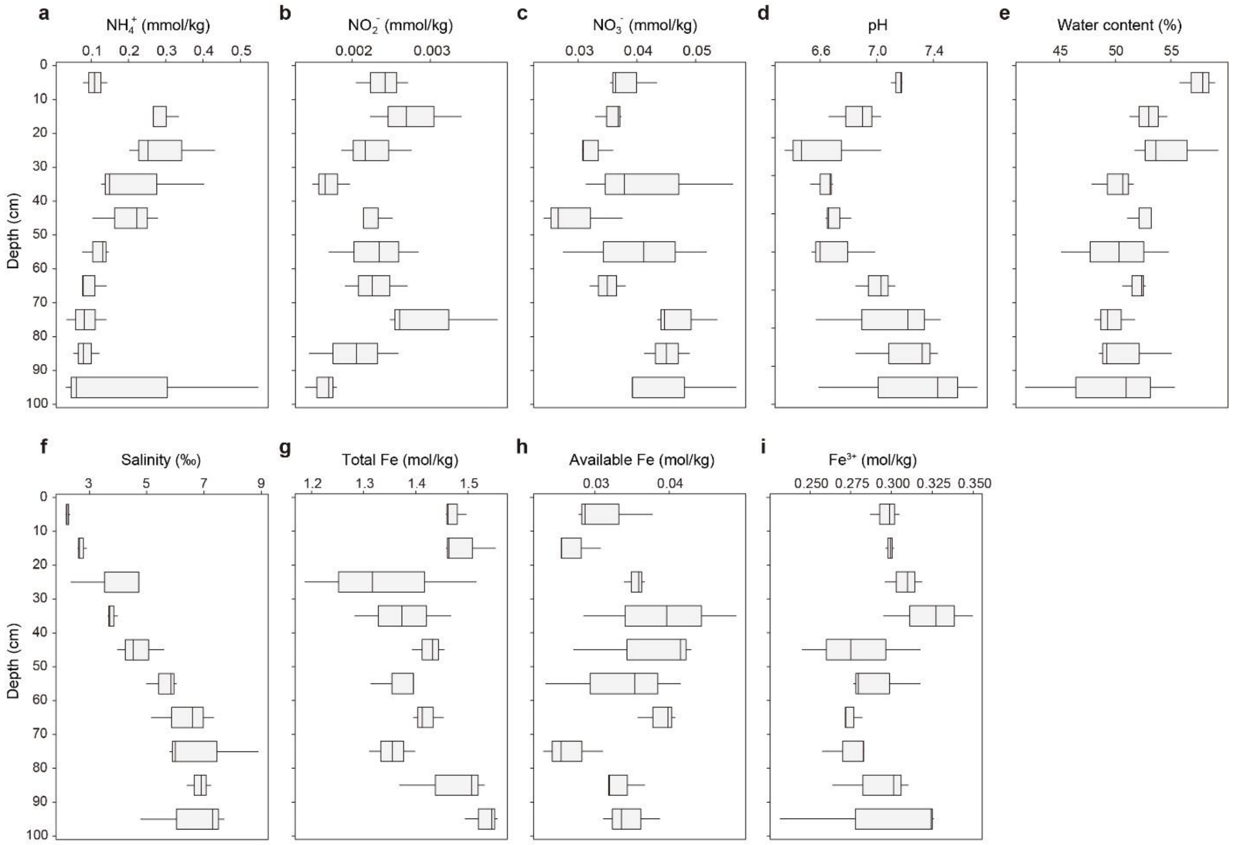

Fig. S3. The in-depth profile of physicochemical properties. The concentration of NH_4^+ (**a**), NO_2^- (**b**), NO_3^- (**c**), pH (**d**), water content (**e**), salinity (**f**), total Fe (**g**), available Fe (**h**) and Fe^{3+} (**i**) across 10 depths of mangrove sediments. Boxplots depict the 25-75% quantile range of the selected measurements, with the centerline depicting the median (50% quantile). Whiskers show the minimum and maximum values.

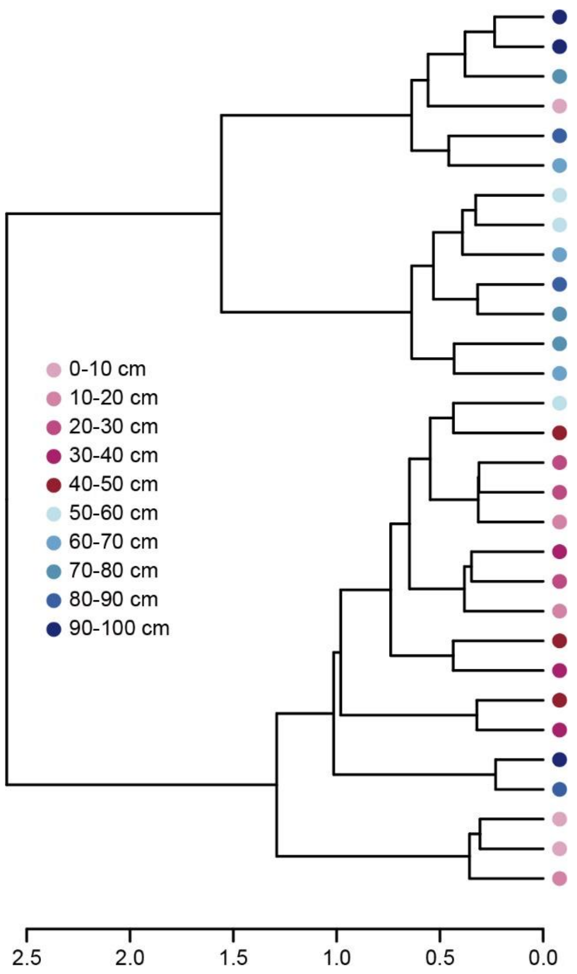

Fig. S4. Bray-Curtis distance-based hierarchical clustering of diazotrophic communities across 10 depths of mangrove sediments. The color gradient represents sediment depth.
